# Ethnobotanical survey of herbal medicines for anti-COVID-19 used by traditional Chinese medicine pharmacies in Taiwan

**DOI:** 10.3389/fphar.2025.1586334

**Published:** 2025-09-03

**Authors:** Min-Han Chi, Chien-Yu Ko, Ting-Wei Chi, Chi-Ruei Huang, Jaung-Geng Lin, Jung Chao, Shyh-Shyun Huang

**Affiliations:** ^1^ School of Pharmacy, China Medical University, Taichung, Taiwan; ^2^ Department of Rehabilitation, Far Eastern Memorial Hospital, New Taipei City, Taiwan; ^3^ School of Medicine, Tzu Chi University, Hualien, Taiwan; ^4^ School of Chinese Medicine, College of Chinese Medicine, China Medical University, Taichung, Taiwan; ^5^ Department of Chinese Pharmaceutical Sciences and Chinese Medicine Resources, Master Program for Food and Drug Safety, Chinese Medicine Research Center, China Medical University, Taichung, Taiwan; ^6^ Department of Food Nutrition and Health Biotechnology, Asia University, Taichung, Taiwan

**Keywords:** SARS-CoV-2, COVID-19, traditional Chinese medicine, ethnobotany, Taiwan

## Abstract

**Introduction:**

Traditional Chinese medicine (TCM) pharmacies in Taiwan have been a vital healthcare resource in Chinese communities, with generations of accumulated TCM knowledge playing a crucial role in infectious disease prevention and treatment. However, as proprietors age and the industry declines, this valuable ethnomedicinal knowledge faces the risk of being lost. Therefore, documenting and analyzing the knowledge used by Taiwanese TCM pharmacies in combating COVID-19 is essential for the preservation and application of ethnomedicine.

**Methods:**

This study employed a stratified sampling method to survey 106 TCM pharmacies in Taiwan, collecting 61 TCM formulae against COVID-19. The medicinal materials were compiled, and analysis were conducted using relative frequency of citation (RFC) and the Phi correlation coefficient to examine the relationships among TCMs. Furthermore, cluster analysis was applied to establish TCM combination patterns.

**Results:**

This study recorded a total of 70 medicinal materials and identified 30 commonly used ones, categorizing them into four major groups: Group A (heat-clearing and detoxifying): As similar as NRICM101, suitable for moderate to severe cases. Group B (heat-clearing and tonifying): Suitable for mild cases, with both antiviral and immune-boosting effects. Group C (symptom relief): Primarily used for relieving cough, expelling phlegm, and alleviating discomfort. Group D (tonifying and strengthening): Suitable for uninfected individuals to enhance immunity.

**Discussion:**

This study successfully documented and analyzed the TCM usage patterns of Taiwanese TCM pharmacies in combating COVID-19, revealing their alignment with modern TCM theories. The findings not only contribute to the preservation of ethnobotanical knowledge but also serve as a reference for developing herbal prevention strategies and healthcare products, ensuring that traditional medical wisdom can play a greater role within the modern healthcare system.

## 1 Introduction

Coronavirus Disease 2019 (COVID-19) first emerged in Wuhan, China, in early December 2019 (Ahn et al., 2020; Zhou P. et al., 2020; Zhu et al., 2020). Research identified the pathogen as a novel coronavirus, named SARS-CoV-2 (Coronaviridae Study Group of the International Committee on Taxonomy of Viruses, 2020). According to statistics from the World Health Organization (WHO), as of 2 February 2025, the global number of confirmed COVID-19 cases has exceeded 770 million, with more than 7 million deaths (World Health Organization, 2024). Although the COVID-19 pandemic has gradually subsided with the increase in vaccination rates, studies have indicated that COVID-19 infection significantly raises the risk of cardiovascular complications and chronic kidney disease (Del Vecchio et al., 2024). Therefore, the search for potential drugs to prevent and treat COVID-19 infection remains important and necessary. According to the COVID-19 treatment guidelines published by the National Institutes of Health (NIH), recommended drugs for non-hospitalized adult patients include glucocorticoids (e.g., Dexamethasone), 3C-like protease inhibitors [e.g., Nirmatrelvir/ritonavir (Paxlovid^®^)], Nucleoside Analogues (e.g., Molnupiravir), and RNA-dependent RNA polymerase inhibitors (e.g., Remdesivir) (COVID-19 Treatment Guidelines Panel, 2024). The most used therapeutic drug in clinical settings is Paxlovid^®^. Although studies have shown that Paxlovid^®^ can reduce the severity of COVID-19 symptoms, its high cost and potential for multiple drug interactions still make it unaffordable and intolerable for many patients with chronic diseases (Marzolini et al., 2022). In addition, the development of other new drugs takes a long time, which is not a quick solution. As a result, there has been an international effort to explore the therapeutic and preventive effects of traditional Chinese herbal medicine for COVID-19.

As countries worldwide strive to control the COVID-19 pandemic, the development of new drugs is time-consuming and cannot provide an immediate solution. Therefore, drug repurposing has become the primary strategy for using existing Western medicines to treat COVID-19 (Singh et al., 2020; Zhou Y. et al., 2020; Asselah et al., 2021; Yousefi et al., 2021). However, the side effects of many Western medicines are often difficult for patients to tolerate, and the therapeutic effects of drug repurposing remain limited. As a result, there has been a growing international effort to explore the therapeutic and preventive effects of traditional Chinese herbal medicine for COVID-19. A previous study tracked 782 patients who were treated with the traditional Chinese herbal formula—Qingfei Paidu Decoction (QFPDD)—for COVID-19. The findings revealed that early use of QFPDD significantly shortened the disease recovery period and hospitalization duration (Shi et al., 2020). Another systematic review found that the disease cure rate in the combined Chinese and Western medicine group was higher than that in the Western medicine-only group. Additionally, it was observed that the integration of Chinese and Western medicine significantly reduced the length of hospital stay for patients (Liu M. et al., 2020). In summary, the use of traditional Chinese herbal medicine alone or in combination with Western medicine can effectively shorten the COVID-19 recovery period. Therefore, exploring potential effective herbal medicines for the prevention and treatment of COVID-19 holds significant promise.

Previous studies have indicated that the annual prevalence of over-the-counter traditional Chinese medicine (TCM) purchases among the Taiwanese population is as high as 74.8% (Hu et al., 2020). According to statistics from the Ministry of Health and Welfare (MOHW) of Taiwan, as of the end of 2020, there were 8,348 TCM pharmacies across Taiwan (Ministry of Health and Welfare, 2024). On average, there are 0.231 TCM pharmacies per square kilometer, making them highly accessible. As a result, TCM pharmacies remain the primary channel for the Taiwanese public to purchase herbal medicines. These pharmacies have existed in Taiwanese society long before the introduction and development of Western medicine, with generations of accumulated TCM knowledge serving as a crucial tool for combating diseases in Chinese communities. Even today, despite the rapid advancement of Western medicine, the knowledge preserved in TCM pharmacies continues to play a vital role in disease prevention and health maintenance within Chinese society. Today, TCM pharmacies in Taiwan serve as rich repositories of TCM knowledge. However, the average age of TCM pharmacy proprietors has exceeded 60 years, and the number of pharmacies is decreasing annually. Therefore, exploring and preserving the extensive TCM knowledge passed down through these pharmacies has become an urgent task.

In this study, we conducted fieldwork to community TCM pharmacies in Taiwan, collecting the herbal formulae they offered for COVID-19 treatment. We analyzed these formulae, identified candidate medicinal materials used against COVID-19, and applied statistical analysis to examine the relationships between these medicinal materials. By doing so, we uncovered the selection patterns of medicinal materials used by TCM pharmacies in combating COVID-19. Ultimately, the findings of this study aim to provide clinical practitioners with reference data for COVID-19 treatment prescriptions and support the development and application of health products.

## 2 Materials and methods

### 2.1 Ethical review

This study was conducted from July 2021 to June 2022 and was approved by the Central Regional Research Ethics Committee of China Medical University and Hospital (Approval No. CMUH110-REC2-115) (Supplementary Figure S1).

### 2.2 Study area and formulae collection

The study area for this research is Taiwan, a spindle-shaped island located in the western Pacific Ocean, positioned between the Republic of the Philippines and the Ryukyu Islands. To the west, Taiwan is separated from the People’s Republic of China by the Taiwan Strait. According to the Taiwan Geodetic Datum 1997 (TWD97) coordinate system, the geographic reference point of Taiwan is (23°58′N, 120°58′E), with a total land area of approximately 36,000 km^2^.

The sampling data for this study were based on the official statistics published by the Department of Statistics at the MOHW in Taiwan (Ministry of Health and Welfare, 2024), using the data on the number of licensed TCM pharmacies operating under Article 103 of the Pharmaceutical Affairs Act as of the end of 2020, and stratified by county and city. The number of sampled pharmacies in each county and city was determined according to the distribution ratio presented in the official statistics (Supplementary Table S1).

**FIGURE 1:**
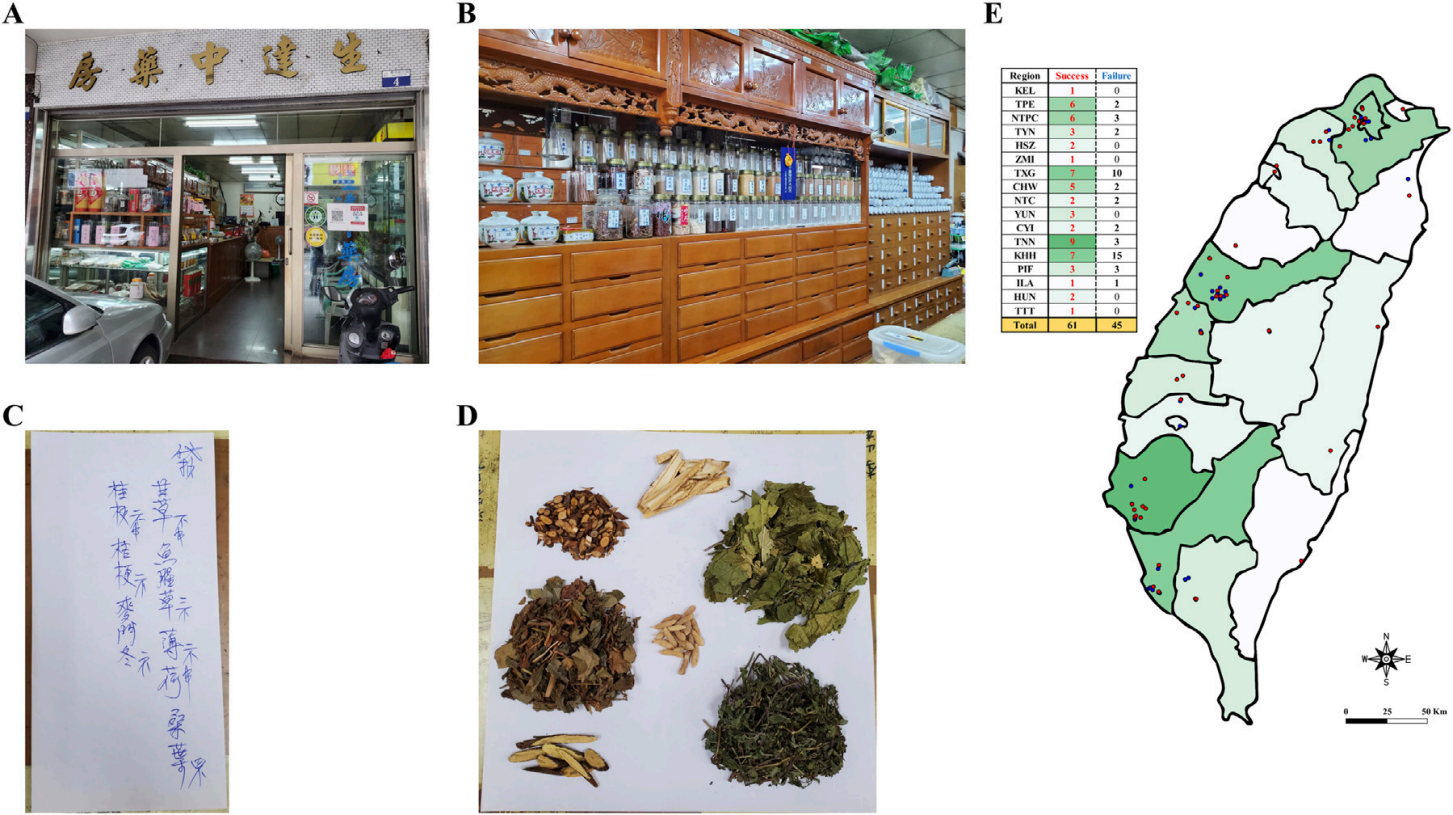
Photograph of TCM pharmacy **(A)** Appearance of TCM pharmacy **(B)** TCM cabinet **(C)** Anti-COVID-19 handwritten prescription **(D)** Anti-COVID-19 formula **(E)** Distribution map of 106 TCM pharmacies. Note: The red dots represent the locations of successful collection pharmacies; the blue dots represent the locations of failed collection pharmacies. KEL, Keelung City; TPE, Taipei City; NTPC, New Taipei City; TYN, Taoyuan City; HSZ, Hsinchu County; ZMI, Miaoli County; TXG, Taichung City; CHW, Changhua County; NTC, Nantou County; YUN, Yunlin County; CYI, Chiayi County; TNN, Tainan City; KHH, Kaohsiung City; PIF, Pingtung County; ILA, Yilan County; HUN, Hualien County; TTT, Taitung County.

After determining the sample size, the pharmacies in each county and city were visited to purchase the anti-COVID-19 formulae and the purchase information was recorded in detail (Figure 1). After reaching the estimated number of formulae to be collected in each county and city, the formulae were taken to the laboratory for disassembly.

### 2.3 Medicinal material identification and information sorting

In this study, the macroscopical identification method was used during formulae disassembly and medicinal materials recognition (Supplementary Figure S2). Firstly, the identification of medicinal materials was conducted through visual, olfactory, gustatory, and tactile examination (Zhao et al., 2011). Secondly, image authentication was carried out against the “Taiwan Herbal Pharmacopeia Database” (Department of Chinese Medicine and Pharmacy, 2024) and the “Color Atlas for the Authentic and Superior Chinese Material Medica” (Xian-Zhe, 2007). Thirdly, the results of the secondary authentication were provided to a review panel consisting of three PhDs in traditional Chinese medicine and two practitioners in Chinese medicine industry. These five experts conducted tertiary authentication by double-checking the results of the secondary authentication, and authentication report was compiled to confirm the correct origin of the medicinal materials. Afterwards, the medicinal materials were then photographed using a smartphone, and detailed information for each formula was compiled into a digital report (PDF file) for recordkeeping. Finally, each formula was ultimately packed into a separate bag, with the purchase location and date handwritten on the bag, and stored at the specimen storage area in the China Medical University.

In order to include information about all the medicinal materials collected in this study, the study referred to the *Taiwan Herbal Pharmacopeia fourth Edition* (Taiwan Herbal Pharmacopeia 4th Edition Committee, 2021), the *Pharmacopoeia of the People’s Republic of China 2020 Edition* (Chinese Pharmacopoeia Commission, 2020), and *Chinese Materia Medica* (National Administration of Traditional Chinese Medicine “Chinese Materia Medica” Editorial Board, 1999) for medicinal material data collection. The collected information included kingdom, family, local name, scientific name, part used, traditional use, and property and flavor. If discrepancies exist among the three references, the hierarchy of information, from highest to lowest authority, is as follows: the *Taiwan Herbal Pharmacopeia fourth Edition*, the *Pharmacopoeia of the People’s Republic of China 2020 Edition*, and *Chinese Materia Medica*. For medicinal materials with differing family classifications and scientific names across different references, verification and standardization were conducted using World Flora Online database (Royal Botanic Gardens and Garden, 2024).

### 2.4 Data analysis

The data collected in this study were analyzed and visualized using Microsoft Office 365 Excel, GraphPad Prism (Version 10.2.2), Adobe Illustrator (Version 28.5.0.132), and RStudio (Version 2024.04.2). For quantitative analysis, two parameters were applied: relative frequency of citation (RFC) and the Phi coefficient. Referring to the RFC formula definition from previous studies (Ahmad et al., 2017), the formula was modified to fit this study, as shown below:

$$\text{RFC}=\frac{\text{the}\;\text{number}\;\text{of}\;\text{one}\;\text{medicinal}\;\text{material}\;\text{mentioned}\;\text{in}\;\text{total}\;\text{formulae}\;}{\text{the}\;\text{total}\;\text{number}\;\text{of}\;\text{collected}\;\text{formulae}}$$

For the Phi coefficient, a contingency table was first created to record the occurrence frequency of any two medicinal materials. Then, referring to the Phi coefficient formula from previous studies (Chytrý et al., 2002; Cui et al., 2016), it was modified to fit this study, as shown below:

$$\text{phi coefficient }\left(\varphi \right)=\frac{\left(a\times d\right)‐\left(b\times c\right)}{\sqrt{\left(a+b\right)\times \left(c+d\right)\times \left(a+c\right)\times \left(b+d\right)}}$$

In this formula, “a” represents the number of formulae in which both TCM-A and TCM-B appear; “b” represents the number of formulae in which TCM-A appears but TCM-B does not; “c” represents the number of formulae in which TCM-A does not appear but TCM-B does; and “d” represents the number of formulae in which neither TCM-A nor TCM-B appear. The Phi coefficient ranges from −1 to +1, with higher values indicating stronger associations between the medicinal materials. The calculated Phi coefficient values were then combined with cluster analysis to generate a network graph, which was used to explore the combination patterns of medicinal materials in Taiwan TCM pharmacies for combating COVID-19.

## 3 Results

### 3.1 Results of anti-COVID-19 formulae and medicinal material information collection

According to the data published by the Department of Statistics at MOHW in Taiwan at the end of 2020, there were a total of 8,348 TCM pharmacies in Taiwan, operating in accordance with Article 103 of the Pharmaceutical Affairs Act. Among these, Kaohsiung City had the highest number of pharmacies, with 1,350, while Taitung County had the fewest, with only 48 (Supplementary Table S1). This study conducted stratified sampling based on counties and cities, with the sample size determined according to the proportion of TCM pharmacies in each area. A total of 106 pharmacies were randomly selected for fieldwork. As a result, 61 anti-COVID-19 formulae were successfully collected. Additionally, 45 pharmacies reported that they did not sell any anti-COVID-19 formula (Figure 1).

Differences in taxonomic methods and the specific versions applied during the compilation of different pharmacopeias have led to inconsistencies in classification. Therefore, for the families and scientific names, this study uses the content recorded in World Flora Online as the standard to correct and integrate the plant information from each pharmacopeia (Royal Botanic Gardens and Garden, 2024). In this study, among the 61 formulae, a total of 70 medicinal materials were identified. Of these, 1 medicinal material belongs to the Fungi kingdom (1.43%), while the remaining 69 medicinal materials belong to the Plantae kingdom (98.57%). These 70 medicinal materials are distributed across 40 families, with the most common families being the Lamiaceae (n = 8, 11.43%), Apiaceae (n = 6, 8.57%), Fabaceae (n = 4, 5.71%), and Compositae (n = 4, 5.71%). Statistical results of the parts used suggested that 17 medicinal parts are used, with the radix being the most common (n = 19, 27.14%), followed by the rhizoma (n = 11, 15.71%) (Supplementary Figure S3).

### 3.2 Properties, flavors, and traditional uses of medicinal materials against COVID-19

**FIGURE 2:**
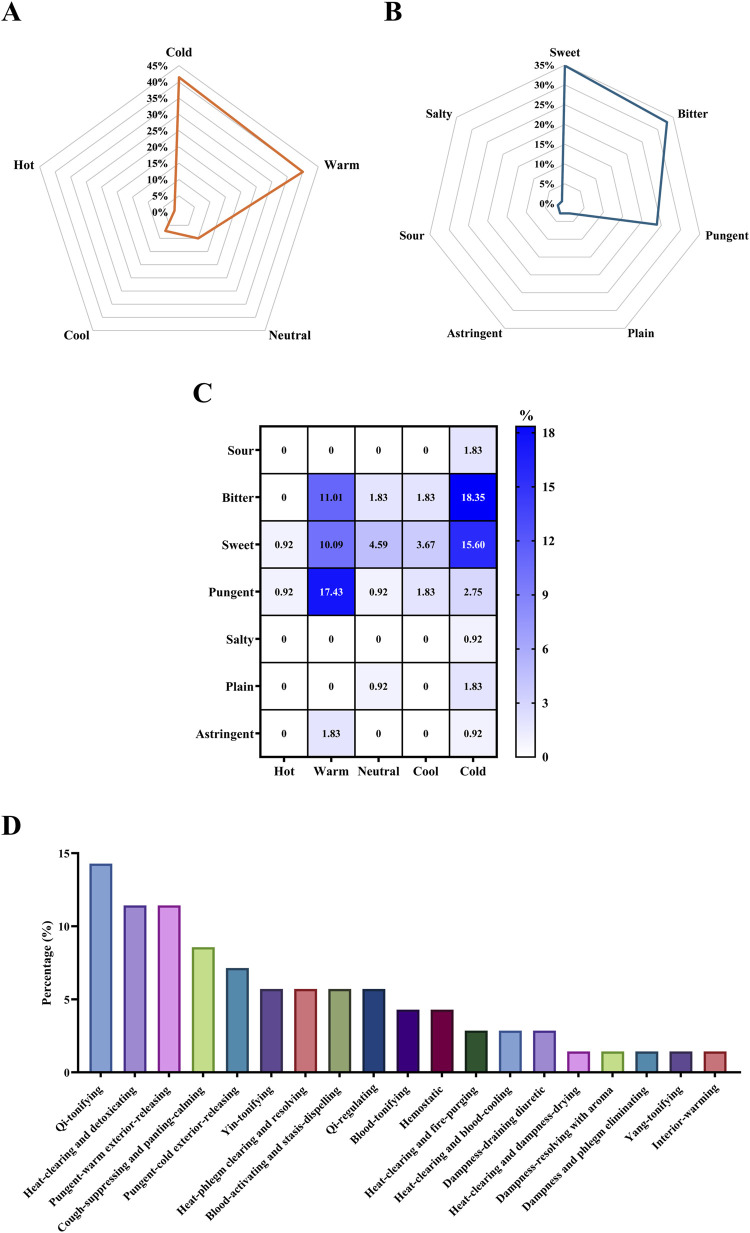
Flavor, property and traditional use statistics analysis charts of 70 medicinal materials combating COVID-19 **(A)** Radar plot of properties **(B)** Radar plot of flavors **(C)** Correlation heat map of flavors and properties **(D)** Bar chart of traditional uses.

Each medicinal material may possess multiple medicinal flavors, but it will only have one medicinal property. Medicinal properties can be classified as hot, warm, neutral, cool, or cold; medicinal flavors can be classified as sour, bitter, sweet, pungent, salty, bland, and astringent. This study consolidates pharmacopeial information for 70 medicinal materials, with cold properties (41.43%) and warm properties (40.00%) being the most prevalent (Figure 2A). The medicinal flavors are predominantly sweet (34.86%), bitter (33.03%), and pungent (23.85%) (Figure 2B). When both medicinal flavors and properties are considered together, the most common combinations are bitter and cold (18.35%), pungent and warm (17.43%), and sweet and cold (15.60%) (Figure 2C). In terms of traditional uses, among the 70 medicinal materials consolidated in this study, the top three most common traditional uses are “Qi-tonifying” (14.29%), “Heat-clearing and detoxicating” (11.43%), and “Pungent-warm exterior-releasing” (11.43%) (Figure 2D).

### 3.3 Correlation of medicinal materials against COVID-19

To determine the usage frequency of each medicinal material, this study calculated the RFC values for the 70 medicinal materials and excluded those with RFC values below 0.05 from the correlation statistics. Among the 70 medicinal materials, 30 had RFC values greater than 0.05 (referred to as commonly used medicinal materials), in descending order of *Glycyrrhiza uralensis* Fisch. (RFC = 0.574), *Houttuynia cordata* Thunb. (RFC = 0.475), *Ziziphus jujuba* Mill. (RFC = 0.459), *Mentha canadensis* L. (RFC = 0.361), *Lycium chinense* Mill. (RFC = 0.361), *Astragalus mongholicus* Bunge (RFC = 0.328), *Hedysarum polybotrys* Hand.-Mazz. (RFC = 0.311), *Morus alba* L. (RFC = 0.279), *Lonicera japonica* Thunb. (RFC = 0.230), *Ophiopogon japonicus* (Thunb.) Ker Gawl. (RFC = 0.197), *Saposhnikovia divaricata* (Turcz.) Schischk. (RFC = 0.180), *Cinnamomum cassia* (L.). J.Presl (RFC = 0.180), *Platycodon grandiflorus* (Jacq.) A.DC. (RFC = 0.180), *Nepeta tenuifolia* Benth. (RFC = 0.164), *Perilla frutescens* (L.). Britton (RFC = 0.148), *Chrysanthemum morifolium* Ramat. (RFC = 0.131), *Codonopsis pilosula* (Franch.) Nannf. (RFC = 0.131), *Atractylodes macrocephala* Koidz. (RFC = 0.115), *Angelica sinensis* (Oliv.) Diels (RFC = 0.115), *Scutellaria baicalensis* Georgi (RFC = 0.098), *Panax quinquefolius* L. (RFC = 0.098), *Zingiber officinale* Roscoe (RFC = 0.098), *Trichosanthes kirilowii* Maxim. (RFC = 0.082), *Isatis tinctoria* L. (RFC = 0.082), *Ligusticum striatum* DC. (RFC = 0.082), *Agastache rugosa* (Fisch. and C.A.Mey.) Kuntze (RFC = 0.082), *Phragmites australis* (Cav.) Trin. ex Steud. (RFC = 0.082), *Panax ginseng* C.A.Mey. (RFC = 0.082), *Strobilanthes cusia* (Nees) Kuntze (RFC = 0.066), and *Citrus reticulata* Blanco (RFC = 0.066) (Table 1).

**TABLE 1:** Information on 70 medicinal materials used in anti-COVID-19 formulae.

No	Latin name	Local name	Scientific name	Code	Family	Part used	RFC	Traditional use	Flavors/Properties	Dosage^a^
1	Glycyrrhiza radix et rhizoma	Kan ts’ao 甘草	*Glycyrrhiza uralensis* Fisch	GLY	Fabaceae	Rhizoma et radix	0.574	Qi-tonifying	Sweet/Neutral	1.5–30 g
2	Houttuyniae herba	Yü hsing ts’ao 魚腥草	*Houttuynia cordata* Thunb	HNH	Saururaceae	Herba	0.475	Heat-clearing and detoxicating	Pungent/Mild cold	3–30 g
3	Jujubae fructus	Ta tsao 大棗	*Ziziphus jujuba* Mill	JJB	Rhamnaceae	Fructus	0.459	Qi-tonifying	Sweet/Warm	2–71 g
4	Menthae herba	Po hê 薄荷	*Mentha canadensis* L	MTH	Lamiaceae	Herba	0.361	Pungent-cold exterior-releasing	Pungent/Cool	1.5–40.5 g
5	Lycii fructus	Kou ch’i tzu 枸杞子	*Lycium chinense* Mill	LCF	Solanaceae	Fructus	0.361	Yin-tonifying	Sweet/Neutral	3.75–45 g
6	Astragali radix	Huang ch’i 黃耆	*Astragalus mongholicus* Bunge	AGR	Fabaceae	Radix	0.328	Qi-tonifying	Sweet/Mild warm	3.75–49 g
7	Hedysari radix	Hung ch’i 紅耆	*Hedysarum polybotrys* Hand.-Mazz	HDR	Fabaceae	Radix	0.311	Qi-tonifying	Sweet/Mild warm	6–40.5 g
8	Mori folium	Sang yeh 桑葉	*Morus alba* L	MRF	Moraceae	Folium	0.279	Pungent-cold exterior-releasing	Sweet, Bitter/Cold	5.75–33.75 g
9	Lonicerae japonicae flos	Chin yin hua 金銀花	*Lonicera japonica* Thunb	LJF	Caprifoliaceae	Flos	0.230	Heat-clearing and detoxicating	Sweet/Cold	1.5–19.5 g
10	Ophiopogonis radix	Mai mên tung 麥門冬	*Ophiopogon japonicus* (Thunb.) Ker Gawl	OPR	Asparagaceae	Radix	0.197	Yin-tonifying	Sweet, Mild bitter/Mild cold	2–15.5 g
11	Saposhnikoviae radix et rhizoma	Fang fêng 防風	*Saposhnikovia divaricata* (Turcz.) Schischk	SKE	Apiaceae	Radix et Rhizoma	0.180	Pungent-warm exterior-releasing	Pungent, Sweet/Mild warm	3–19 g
12	Cinnamomi ramulus	Kuei chih 桂枝	*Cinnamomum cassia* (L.) J.Presl	CMR	Lauraceae	Ramulus	0.180	Pungent-warm exterior-releasing	Pungent, Sweet/Warm	2–61.875 g
13	Platycodonis radix	Chieh kêng 桔梗	*Platycodon grandiflorus* (Jacq.) ADC.	PCR	Campanulaceae	Radix	0.180	Heat-phlegm clearing and resolving	Bitter, Pungent/Neutral	1–20 g
14	Nepetae herba	Ching chieh 荊芥	*Nepeta tenuifolia* Benth	NPH	Lamiaceae	Herba	0.164	Pungent-warm exterior-releasing	Pungent/Mild warm	3–17 g
15	Perillae folium	Tzu su yeh 紫蘇葉	*Perilla frutescens* (L.) Britton	PLF	Lamiaceae	Folium	0.148	Pungent-warm exterior-releasing	Pungent/Warm	3.75–15 g
16	Chrysanthemi flos	Chü hua 菊花	*Chrysanthemum morifolium* Ramat	CSF	Compositae	Flos	0.131	Pungent-cold exterior-releasing	Sweet, Bitter/Mild cold	3.5–10.5 g
17	Codonopsis radix	Tang san 黨參	*Codonopsis pilosula* (Franch.) Nannf	CSR	Campanulaceae	Radix	0.131	Qi-tonifying	Sweet/Neutral	3.75–22 g
18	Atractylodis macrocephal rhizoma	Pai chu 白朮	*Atractylodes macrocephala* Koidz	AMR	Compositae	Rhizoma	0.115	Qi-tonifying	Bitter, Sweet/Warm	1–16.5 g
19	Angelicae sinensis radix	Tang kuei 當歸	*Angelica sinensis* (Oliv.) Diels	ASR	Apiaceae	Radix	0.115	Blood-tonifying	Sweet, Pungent/Warm	3–15 g
20	Scutellapiae radix	Huang ch’in 黃芩	*Scutellaria baicalensis* Georgi	STR	Lamiaceae	Radix	0.098	Heat-clearing and dampness-drying	Bitter/Cold	2–12.5 g
21	Panacis quinquefolii radix	Hsi yang shên 西洋參	*Panax quinquefolius* L	PQR	Araliaceae	Radix	0.098	Qi-tonifying	Sweet, Mild bitter/Cool	3–21 g
22	Zingiberis rhizoma recens	Shêng chiang 生薑	*Zingiber officinale* Roscoe	ZGB	Zingiberaceae	Rhizoma	0.098	Pungent-warm exterior-releasing	Pungent/Warm	2–30 g
23	Trichosanthis fructus	Kua lou 栝蔞	*Trichosanthes kirilowii* Maxim	TSF	Cucurbitaceae	Fructus	0.082	Heat-phlegm clearing and resolving	Sweet, Mild bitter/Cold	4–20.5 g
24	Isatidis radix	Pei pan lan kên 北板藍根	*Isatis tinctoria* L	ITR	Brassicaceae	Radix	0.082	Heat-clearing and detoxicating	Bitter/Cold	1–11 g
25	Chuanxiong rhizoma	Ch’uan ch’iung 川芎	*Ligusticum striatum* DC.	CXR	Apiaceae	Rhizoma	0.082	Blood-activating and stasis-dispelling	Pungent/Warm	4.7–11.25 g
26	Agastachis herba	Huo hsiang 藿香	*Agastache rugosa* (Fisch. and C.A.Mey.) Kuntze	ATH	Lamiaceae	Herba	0.082	Dampness-resolving with aroma	Pungent/Mild warm	12–20 g
27	Phragmitis rhizoma	Lu kên 蘆根	*Phragmites australis* (Cav.) Trin. ex Steud	PMR	Poaceae	Rhizoma	0.082	Heat-clearing and fire-purging	Sweet/Cold	3–8.67 g
28	Ginseng radix et rhizoma	Jên shên 人參	*Panax ginseng* C.A.Mey	GSG	Araliaceae	Radix et Rhizoma	0.082	Qi-tonifying	Sweet, Mild bitter/Mild warm	3.75–8.5 g
29	Strobilanthii cusiae rhizoma et radix	Nan pan lan kên 南板藍根	*Strobilanthes cusia* (Nees) Kuntze	SCR	Acanthaceae	Rhizoma et radix	0.066	Heat-clearing and detoxicating	Bitter/Cold	10.5–21 g
30	Citri reticulatae pericarpium	Chü p’i 橘皮	*Citrus reticulata* Blanco	CRP	Rutaceae	Pericarpium	0.066	Qi-regulating	Bitter, Pungent/Warm	2–13.5 g
31	Salviae miltiorrhizae radix et rhizoma	Tan shên 丹參	*Salvia miltiorrhiza* Bunge	SME	Lamiaceae	Radix et rhizoma	0.049	Blood-activating and stasis-dispelling	Bitter/Mild cold	8–18.75 g
32	Glehniae radix	Pei sha shên 北沙參	*Glehnia littoralis* F.Schmidt ex Miq	GNR	Apiaceae	Radix	0.049	Yin-tonifying	Sweet, Mild bitter/Mild cold	10–21 g
33	Bupleuri radix	Ch’ai hu 柴胡	*Bupleurum chinense* DC.	BPR	Apiaceae	Radix	0.049	Pungent-cold exterior-releasing	Pungent/Bitter/Mild cold	4–12.5 g
34	Puerariae radix	Kê kên 葛根	*Pueraria montana* var. *chinensis* (Ohwi) Sanjappa and Pradeep	PRR	Fabaceae	Radix	0.049	Pungent-cold exterior-releasing	Sweet, Pungent/Cool	0.493–2 g
35	Armeniacae semen amarum	K’u hsing jên 苦杏仁	*Prunus sibirica* L	ASA	Rosaceae	Semen amarum	0.049	Cough-suppressing and panting-calming	Bitter/Warm	3.75–11.5 g
36	Magnolia cortex	Hou p’o 厚朴	*Magnolia officinalis* Rehder and E.H.Wilson	MNC	Magnoliaceae	Cortex	0.033	Qi-regulating	Bitter, Pungent/Warm	3–8.5 g
37	Angeliga dahurica radix	Pai chih 白芷	*Angelica dahurica* (Hoffm.) Benth. and Hook.f. ex Franch. and Sav	ADR	Apiaceae	Radix	0.033	Pungent-warm exterior-releasing	Pungent/Warm	9–12.5 g
38	Lilii bulbus	Pai hê 百合	*Lilium lancifolium* Thunb	LBS	Liliaceae	Bulbus	0.033	Yin-tonifying	Sweet/Mild cold	9.5 g
39	Fritillariae thunbergii bulbus	Chê pei mu 浙貝母	*Fritillaria thunbergii* Miq	FTB	Liliaceae	Bulbus	0.033	Heat-phlegm clearing and resolving	Bitter/Cold	8–17.5 g
40	Poria	Fu ling 茯苓	*Poria cocos* (Schwein.) F.A. Wolf	POR	Polyporaceae	Poria	0.033	Dampness-draining diuretic	Sweet, Plain/Neutral	1–18.5 g
41	Forsythiae fructus	Lien ch’iao 連翹	*Forsythia suspensa* (Thunb.) Vahl	FSR	Oleaceae	Fructus	0.033	Heat-clearing and detoxicating	Bitter/Mild cold	7.5–8 g
42	Perillae caulis	Tzu su kêng 紫蘇梗	*Perilla frutescens* (L.) Britton	PLC	Lamiaceae	Caulis	0.033	Qi-regulating	Pungent/Warm	12.5–19 g
43	Siraitiae fructus	Lo han kuo 羅漢果	*Siraitia grosvenorii* (Swingle) C.Jeffrey ex A.M.Lu and Zhi.Y.Zhang	STF	Cucurbitaceae	Fructus	0.033	Cough-suppressing and panting-calming	Sweet/Cool	16–25 g
44	Taraxaci herba	P’u kung ying 蒲公英	*Taraxacum mongolicum* Hand.-Mazz	TXH	Compositae	Herba	0.016	Heat-clearing and detoxicating	Bitter, Sweet/Cold	18.5 g
45	Dioscoreae rhizoma	Shan yao 山藥	*Dioscorea oppositifolia* L	DSR	Dioscroeaceae	Rhizoma	0.016	Qi-tonifying	Sweet/Neutral	3 g
46	Pinelliae rhizoma	Pan hsia 半夏	*Pinellia ternata* (Thunb.) Makino	PLR	Araceae	Rhizoma	0.016	Dampness and phlegm eliminating	Pungent/Warm	9 g
47	Scrophulariae radix	Hsüan shên 玄參	*Scrophularia ningpoensis* Hemsl	SPR	Scrophulariaceae	Radix	0.016	Heat-clearing and blood-cooling	Sweet, Bitter, Salty/Mild cold	7.5 g
48	Bletillae rhizoma	Pai chi 白及	*Bletilla striata* (Thunb.) Rchb.f	BTR	Orchidaceae	Rhizoma	0.016	Hemostatic	Bitter, Sweet, Astringent/Mild cold	7.5 g
49	Paeoniae radix alba	Pai shao 白芍	*Paeonia lactiflora* Pall	PRA	Paeoniaceae	Radix	0.016	Blood-tonifying	Bitter, Sour/Mild cold	6 g
50	Imperatae rhizoma	Pai mao kên 白茅根	*Imperata cylindrica* (L.) Raeusch	IPR	Poaceae	Rhizoma	0.016	Hemostatic	Sweet/Cold	8 g
51	Stemonae radix	Pai pu 百部	*Stemona sessilifolia* (Miq.) Miq	SNR	Stemonaceae	Radix	0.016	Cough-suppressing and panting-calming	Sweet, Bitter/Mild warm	9.5 g
52	Cinnamomi cortex	Jou kuei 肉桂	*Cinnamomum cassia* (L.) J.Presl	CMC	Lauraceae	Cortex	0.016	Interior-warming	Pungent, Sweet/Highly hot	3 g
53	Reynoutriae multiflorae radix	Hê shou wu 何首烏	*Reynoutria multiflora* (Thunb.) Moldenke	RMR	Polygonaceae	Radix	0.016	Blood-tonifying	Bitter, Sweet, Astringent/Mild warm	20 g
54	Eucommiae cortex	Tu chung 杜仲	*Eucommia ulmoides* Oliv	EMC	Eucommiaceae	Cortex	0.016	Yang-tonifying	Sweet/Warm	18 g
55	Acanthopanacis senticosi radix et rhizoma seu caulis	Tz’u wu chia 刺五加	*Eleutherococcus senticosus* (Rupr. and Maxim.) Maxim	ASE	Araliaceae	Radix et Rhizoma seu Caulis	0.016	Qi-tonifying	Pungent, Mild bitter/Warm	16 g
56	Eriobotryae folium	P’i p’a yeh 枇杷葉	*Eriobotrya japonica* (Thunb.) Lindl	EBF	Rosaceae	Folium	0.016	Cough-suppressing and panting-calming	Bitter/Mild cold	9 g
57	Aurantii fructus Immaturus	Chih shih 枳實	*Citrus × aurantium* L	AFI	Rutaceae	Fructus immaturus	0.016	Qi-regulating	Bitter, Pungent, Sour/Mild cold	6 g
58	Scaphii semen	P’ang ta hai 胖大海	*Scaphium affine* (Mast.) Pierre	SPS	Malvaceae	Semen	0.016	Heat-phlegm clearing and resolving	Sweet, Cold	19.5 g
59	Helminthostachydis radix et rhizoma	Tao ti wu kung 倒地蜈蚣	*Helminthostachys zeylanica* (L.) Hook	HCE	Ophioglossaceae	Radix et Rhizoma	0.016	Heat-clearing and detoxicating	Sweet, Bitter/Cool	18.75 g
60	Iris rhizoma	Yeh kan 射干	*Iris domestica* (L.) Goldblatt and Mabb	ISR	Iridaceae	Rhizoma	0.016	Heat-clearing and detoxicating	Bitter/Cold	14 g
61	Mori cortex	Sang pai p’i 桑白皮	*Morus alba* L	MRC	Moraceae	Cortex	0.016	Cough-suppressing and panting-calming	Sweet/Cold	18.5 g
62	Lophatheri herba	Tan chu yeh 淡竹葉	*Lophatherum gracile* Brongn	LOP	Poaceae	Herba	0.016	Heat-clearing and fire-purging	Sweet, Plain/Cold	4.4 g
63	Asari radix	Hsi hsin 細辛	*Asarum heterotropoides* F.Schmidt	ARR	Aristolochiaceae	Radix	0.016	Pungent-warm exterior-releasing	Pungent/Warm	2 g
64	Nelumbinis folium	Hê yeh 荷葉	*Nelumbo nucifera* Gaertn	NBF	Nymphaeaceae	Folium	0.016	Hemostatic	Bitter/Neutral	11.6 g
65	Ephedrae herba	Ma huang 麻黃	*Ephedra sinica* Stapf	EDH	Ephedraceae	Herba	0.016	Pungent-warm exterior-releasing	Pungent, Mild bitter/Warm	6.5 g
66	Asteris radix et rhizoma	Tzu wan 紫菀	*Aster tataricus* L.f	ARE	Compositae	Radix et Rhizoma	0.016	Cough-suppressing and panting-calming	Pungent, Bitter/Warm	5 g
67	Rehmanniae radix	Ti huang 地黃	*Rehmannia glutinosa* (Gaertn.) DC.	RNR	Plantaginaceae	Radix	0.016	Heat-clearing and blood-cooling	Sweet, Bitter/Cold	6.875 g
68	Alismatis rhizoma	Tsê hsieh 澤瀉	*Alisma plantago-aquatica subsp. orientale* (Sam.) Sam	ALI	Alismataceae	Rhizoma	0.016	Dampness-draining diuretic	Sweet, Plain/Cold	4 g
69	Lycopi herba	Tsê lan 澤蘭	*Lycopus lucidus* var. *hirtus* Regel	LPH	Lamiaceae	Herba	0.016	Blood-activating and stasis-dispelling	Bitter, Pungent/Mild warm	15.8 g
70	Curcumae longae rhizoma	Chiang huang 薑黃	*Curcuma longa* L	CLR	Zingiberaceae	Rhizoma	0.016	Blood-activating and stasis-dispelling	Pungent, Bitter/Warm	7 g

^a^ The dosage range is from the minimum to the maximum of formulae.

**FIGURE 3:**
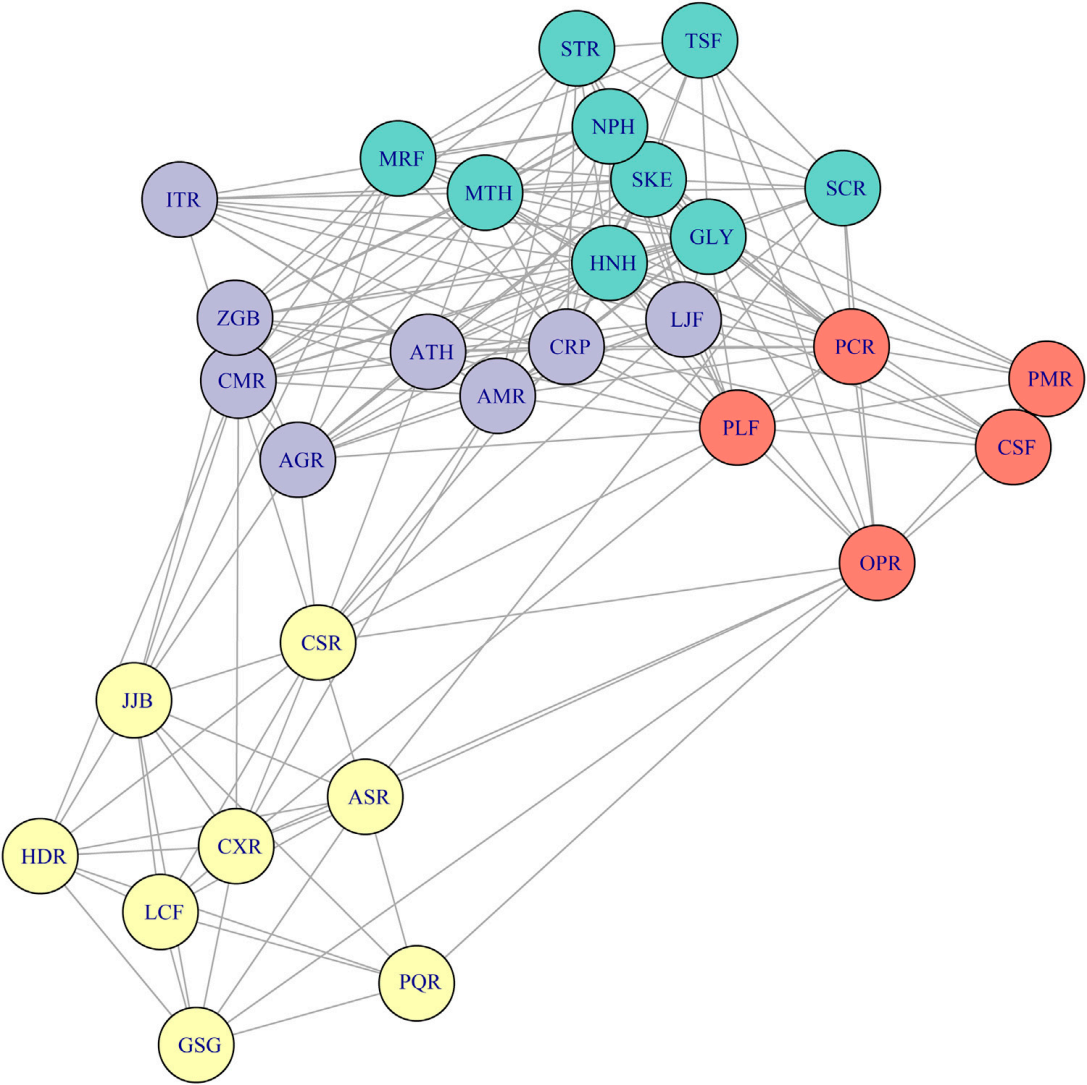
Network diagram of 30 commonly used medicinal materials against COVID-19. Note, The lines indicate a positive Phi coefficient between two medicinal materials. Different colors represent different groups in the cluster analysis. AGR, *A. mongholicus*; AMR, *A. macrocephala*; ASR, *A. sinensis*; ATH, *A. rugosa*; CMR, *C. cassia*; CRP, *C. reticulata*; CSF, *C. morifolium*; CSR, *C. pilosula*; CXR, *L. striatum*; GLY, *Glycyrrhiza uralensis*; GSG, *P. ginseng*; HDR, *H. polybotrys*; HNH, *H. cordata*; ITR, *Isatis tinctoria*; JJB, *Z. jujuba*; LCF, *L. chinense*; LJF, *L. japonica*; MRF, *M. alba*; MTH, *M. canadensis*; NPH, *Nepeta tenuifolia*; OPR, *Ophiopogon japonicus*; PCR, *P. grandiflorus*; PLF, *P. frutescens*; PMR, *P. australis*; PQR, *P. quinquefolius*; SCR, *S. cusia*; SKE, *S. divaricata*; STR, *S. baicalensis*; TSF, *Trichosanthes kirilowii*; ZGB, *Z. officinale*.

The Phi coefficient is a parameter used to measure the correlation between two binary variables. In this study, the occurrence of medicinal materials is considered a binary variable (either present or not present). To clarify the correlation between any two medicinal materials, this study calculates the occurrence of any two medicinal materials among the 30 commonly used medicinal materials in 61 medicinal formulae, constructs a frequency contingency table (Supplementary Table S2), and then calculates the Phi coefficient based on the results of the contingency table. Next, the Phi coefficient was combined with cluster analysis to create a network diagram (Figure 3). The results of the cluster analysis divided the 30 commonly used medicinal materials into four groups: Group A (N = 9) included *G. uralensis*, *H. cordata*, *M. canadensis*, *M. alba*, *S. divaricata*, *N. tenuifolia*, *S. baicalensis*, *T. kirilowii*, and *S. cusia*; Group B (N = 8) included *A. mongholicus*, *L. japonica*, *C. cassia*, *A. macrocephala*, *Z. officinale*, *I. tinctoria*, *A. rugosa*, and *C. reticulata*; Group C (N = 5) included *O. japonicus*, *P. grandiflorus*, *P. frutescens*, *C. morifolium*, and *P. australis*; Group D (N = 8) included *Z. jujuba*, *L. chinense*, *H. polybotrys*, *C. pilosula*, *A. sinensis*, *P. quinquefolius*, *L. striatum*, and *P. ginseng*.

### 3.4 Dosage of medicinal materials against COVID-19

**FIGURE 4:**
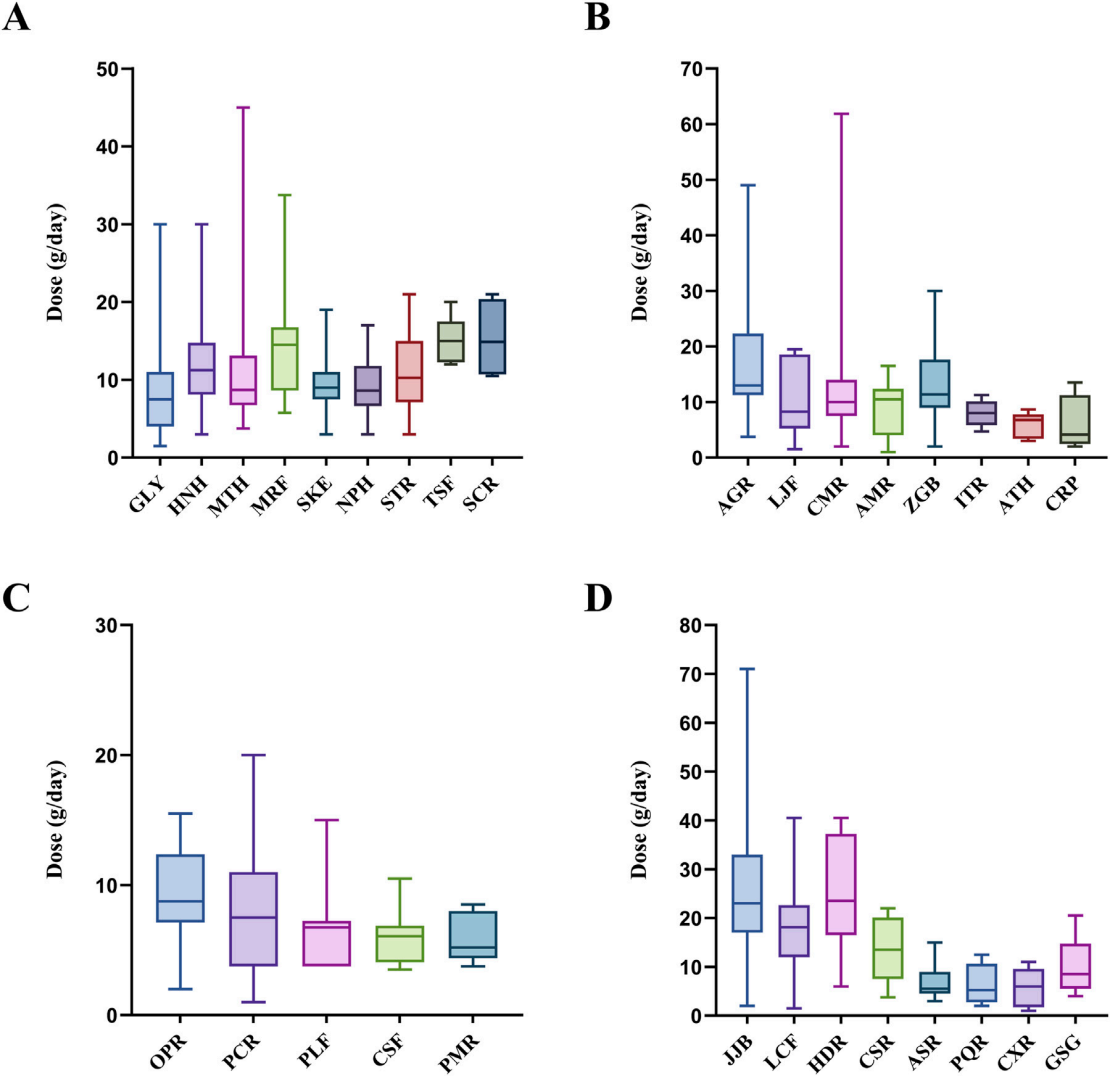
Boxplot of dosages for commonly used medicinal materials against COVID-19 **(A)** Group A (N = 9) **(B)** Group B (N = 8) **(C)** Group C (N = 5) **(D)** Group D (N = 8). AGR, *A. mongholicus*; AMR, *A. macrocephala*; ASR, *A. sinensis*; ATH, *A. rugosa*; CMR, *C. cassia*; CRP, *C. reticulata*; CSF, *C. morifolium*; CSR, *C. pilosula*; CXR, *L. striatum*; GLY, *Glycyrrhiza uralensis*; GSG, *P. ginseng*; HDR, *H. polybotrys*; HNH, *H. cordata*; ITR, *Isatis tinctoria*; JJB, *Z. jujuba*; LCF, *L. chinense*; LJF, *L. japonica*; MRF, *M. alba*; MTH, *M. canadensis*; NPH, *Nepeta tenuifolia*; OPR, *Ophiopogon japonicus*; PCR, *P. grandiflorus*; PLF, *P. frutescens*; PMR, *P. australis*; PQR, *P. quinquefolius*; SCR, *S. cusia*; SKE, *S. divaricata*; STR, *S. baicalensis*; TSF, *Trichosanthes kirilowii*; ZGB, *Z. officinale*.

In this study, based on the calculation results of Louvain community detection model, the 30 commonly used medicinal materials against COVID-19 were divided into four groups (Figure 4). In Group A, the dosage distribution ranged from 1.5 g to 45.0 g. The highest average dosage was *S. cusia* (15.3 g), while the lowest average dosage was *N. tenuifolia* (9.3 g). The medicinal material with the largest dosage range was *M. canadensis* (3.8 g–45.0 g). In Group B, the dosage distribution ranged from 1.0 g to 61.9 g. The highest average dosage was *A. mongholicus* (17.9 g), while the lowest average dosage was *A. rugosa* (5.8 g). The medicinal material with the largest dosage range was *C. cassia* (2.0 g–61.9 g). In Group C, the dosage distribution ranged from 1.0 g to 20.0 g. The highest average dosage was *O. japonicus* (9.3 g), while the lowest average dosage was *P. australis* (6.0 g). The medicinal material with the largest dosage range was *P. grandiflorus* (1.0 g–20.0 g). In Group D, the dosage distribution ranged from 1.0 g to 71.0 g. The highest average dosage was *Z. jujuba* (25.5 g), while the lowest average dosage was *L. striatum* (5.7 g). The medicinal material with the largest dosage range was *Z. jujuba* (2.0 g–71.0 g).

## 4 Discussion

TCM pharmacies existed even before the introduction of Western medicine in Taiwan. Over generations, they have accumulated countless folk formulae and TCM knowledge. These formulae were practiced during early medical deprivation in Taiwan, serving as pioneers of evidence-based medicine. This highlights that TCM pharmacies have become a vital repository for the ethnobotanical knowledge in Taiwan. However, as the older generation of owners ages and the younger generation is reluctant to take over, this valuable ethnobotanical knowledge is gradually disappearing. Therefore, documenting and preserving the folk formulae and TCM selection strategies accumulated by these pharmacies has become a key issue in the preservation of ethnobotanical knowledge in Taiwan.

The rapid global spread of the COVID-19 pandemic and the slow pace of new drug development have made the repurposing of existing medications, along with the use of Chinese herbal medicine and folk formulae, a feasible alternative solution (Fan et al., 2020). In this study, the most widely distributed plant families among the 70 medicinal materials were Lamiaceae, Apiaceae, and Fabaceae. Previous studies have mentioned that compounds such as terpenes, phenylpropanoids, and alkaloids can inhibit viral spike proteins, thus combating coronavirus infections (Babaeekhou et al., 2021; Boozari and Hosseinzadeh, 2021; Ma et al., 2021; Wang et al., 2022; Yi et al., 2022). These three plant families are rich in various terpenes, phenylpropanoids, and alkaloids, which may provide multi-target inhibition of viral infections (Kulkarni et al., 2020; Boozari and Hosseinzadeh, 2021; Liana and Phanumartwiwath, 2022). Furthermore, due to the uneven distribution of effective compounds within the plant, the choice of part used varies (Masumbu et al., 2023). In this study, the majority of the 70 medicinal materials belong to the radix and rhizoma. While the radix and rhizoma may contain the highest concentration of active compounds, harvesting them often results in the death of the plant, which is not in line with the United Nations’ Sustainable Development Goal—Life on Land (Department of Economic and Social Affairs, 2025). Therefore, future research should focus on studying the distribution of effective compounds within plants to identify alternative part used that can support sustainable resource development.

According to previous studies, common clinical manifestations of COVID-19 include fever, cough, systemic inflammatory and sputum production (An et al., 2021). The treatment strategy for COVID-19 in TCM focuses on expelling pathogenic factors and strengthening the body’s vitality (Wang et al., 2020). The former refers to eliminating pathogens and the inflammation they cause, often using heat-clearing and exterior-dispersing medicinal materials; the latter aims to enhance immunity, prevent and treat pathogen invasion, commonly through tonifying medicinal materials. In this study, the 70 medicinal materials are primarily characterized by bitter-cold, pungent-warm, and sweet-cold properties, with traditional uses focused on Qi-tonifying, heat clearing and detoxifying, and dispersing exterior pathogens. According to previous research, sweet-tasting medicinal materials are often rich in glycosides and amino acids, which mainly function to clear heat and tonify. Bitter-cold medicinal materials typically contain alkaloids and primarily serve to clear heat, with significant anti-inflammatory effects. Pungent-tasting medicinal materials contain terpenes and are known for their exterior-dispersing properties (Tie-jun and Liu, 2015; Wang et al., 2018). Other studies have found that bitter-cold medicinal materials can exert anti-inflammatory and angiogenic effects by inhibiting the NF-κB pathway and regulating IL1B, VEGF, and TNF (Zhang et al., 2013). The glycoside components found in sweet-tasting medicinal materials have shown immune-modulating effects in modern studies (Zhang Jing-ya et al., 2016). In summary, the selection of bitter-cold, pungent, and sweet medicinal materials by TCM pharmacies for anti-inflammatory and immune-regulating purposes is a rational combination for combating COVID-19.

**FIGURE 5:**
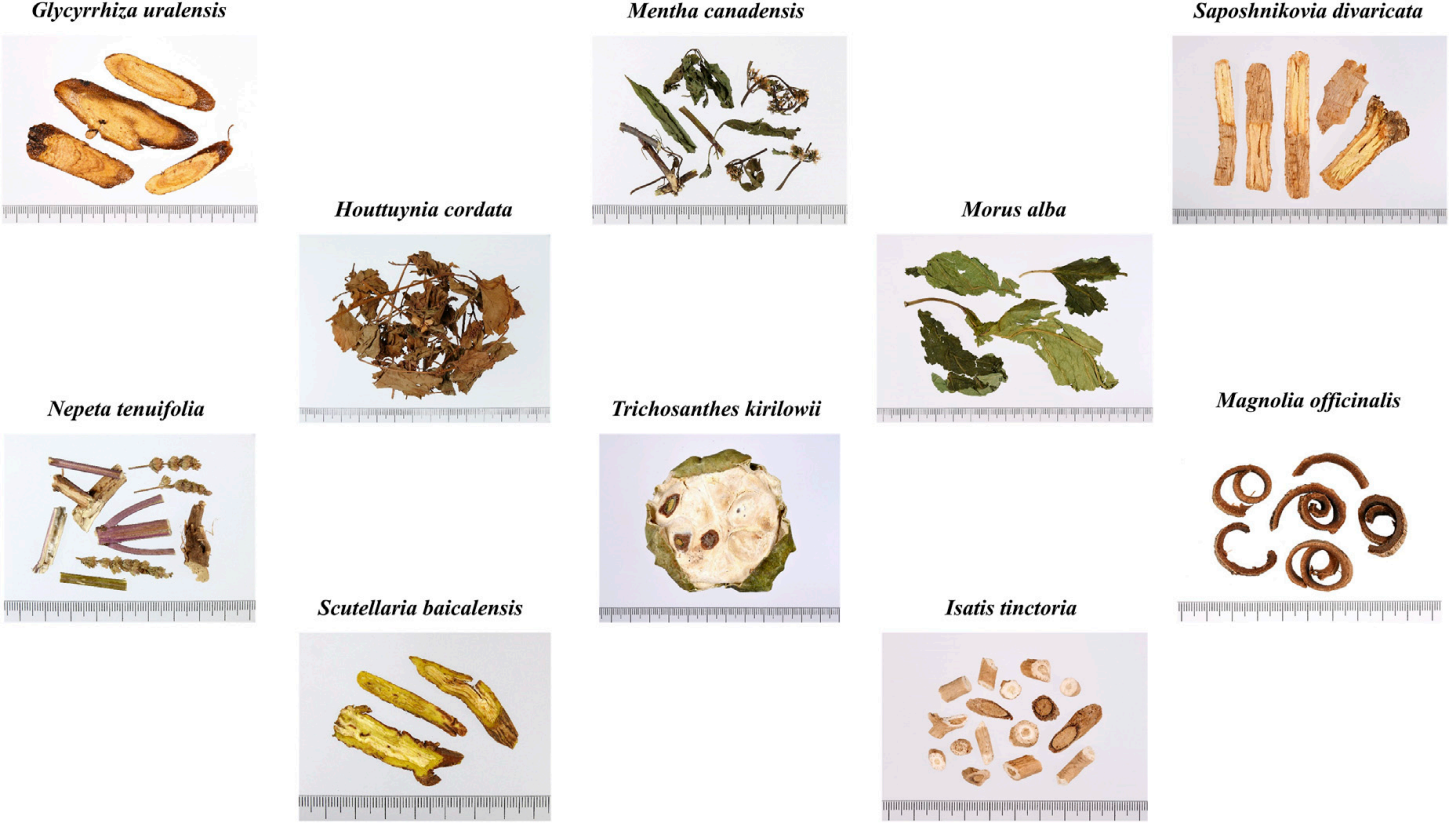
Photograph of *NRICM101* medicinal material composition.

The Louvain community detection model uses modularity to partition network nodes into communities with high internal cohesion and low external connections. It employs a hierarchical clustering method to gradually merge nodes, improving accuracy and efficiency (Traag et al., 2019). In this study, medicinal materials with an RFC greater than 0.05 were defined as commonly used medicinal materials. Each commonly used medicinal material represents one node, the number of positive Phi coefficient between any two medicinal materials is created as edges, and the Phi coefficient value is defined as edge weight in the Louvain community detection model. During the calculation, nodes were aggregated into communities to identify common medicinal material combinations used in TCM pharmacies for combating COVID-19. The results of this study indicate that the medicinal material combinations used in TCM pharmacies for combating COVID-19 can be categorized into four groups: Group A-D. Group A consists of 9 medicinal materials, corresponding to the formula *NRICM101* (Figure 5) [Composition: *G*. *uralensis* (7.5 g), *H*. *cordata* (18.75 g), *M*. *canadensis* (11.25 g), *M*. *alba* (11.25 g), *S*. *divaricata* (7.5 g), *N*. *tenuifolia* (11.25 g), *S*. *baicalensis* (11.25 g), *T*. *kirilowii* (18.75 g), *I*. *tinctoria* (18.75 g), and *Magnolia officinalis* (11.25 g)]. Previous research has shown that *NRICM101* has multi-target effects such as inhibiting the coronavirus spike protein, counteracting immune storms, and blocking viral replication, confirmed by assays such as the inhibition of 3CL protease, SARS-CoV-2 infection, and cytokine inhibition (Tsai et al., 2021). In addition, some studies have shown that *NRICM101* shortened the hospitalization duration, reduced the duration of COVID-19 positive status, and decreased the number of days on mechanical ventilation in initially severe cases (Tsai et al., 2021; Chang et al., 2024). This study found that TCM pharmacies tend to use lower average dosages of each medicinal material compared to *NRICM101*. A possible reason is that the Group A contains many cold property medicinal materials (*H*. *cordata*, *M*. *canadensis*, *M*. *alba*, *S*. *baicalensis*, *T*. *kirilowii*, and *S*. *cusia*). Previous studies have suggested that *NRICM101* may significantly increase the incidence of diarrhea (Chang et al., 2024), and excessive use of cold property medicinal materials can lead to cold-related symptoms such as diarrhea, cold limbs, and loss of appetite (Liu J. et al., 2020). Therefore, it is hypothesized that TCM pharmacies may reduce the dosage to minimize the occurrence of these side effects. Group B consists of 8 medicinal materials, with four herbs categorized as heat-clearing and exterior-releasing agents (*L*. *japonica*, *C*. *cassia*, *Z*. *officinale*, and *I*. *tinctoria*), and two herbs classified as tonifying agents (*A*. *mongholicus* and *A*. *macrocephala*). The selection of these medicinal materials aligns with TCM treatment strategies for COVID-19 (Wang et al., 2020). Previous studies have indicated that *L*. *japonica*, *I*. *tinctoria*, and *A*. *mongholicus* can inhibit M protein and spike protein on SARS-CoV-2, preventing viral entry into cells and replication (Mandal et al., 2021; Yeh et al., 2021). Meanwhile, *C*. *cassia*, *Z*. *officinale*, *A*. *rugosa*, and *C*. *reticulata* are rich in volatile compounds such as terpenes and phenolic compounds, which have been confirmed to possess antiviral activity in various studies (Astani et al., 2010; Wink, 2020; Elsebai and Albalawi, 2022). In addition, some studies have shown that *Z*. *officinale* can accelerate clinical recovery in mild and moderate COVID-19 cases and reduce pulmonary infiltrates (Singh et al., 2023; Ameri et al., 2024). Moreover, when considering the average dosages of the 8 medicinal materials in Group B, it was found that the dosage of *C*. *cassia* is higher than the standard dosage specified in the *Taiwan Herbal Pharmacopeia fourth Edition*, while the dosage of *I*. *tinctoria* is lower than the standard dosage (Taiwan Herbal Pharmacopeia 4th Edition Committee, 2021). Group C consists of five medicinal materials, all of which are symptomatic treatments. Medicinal materials such as *O*. *japonicus*, *P*. *grandiflorus*, *P*. *frutescens*, and *C*. *morifolium* are known for their antitussive, expectorant, and antipyretic effects (Zhang et al., 2015; Chen et al., 2016; Ahmed, 2018; Liu et al., 2024). *Phragmites australis* has been clinically proven to shorten the duration of fever in patients (Fang et al., 2024). In addition, previous study has shown that *P*. *frutescens* extract not only inhibits SARS-CoV-2 replication but also exhibits a synergistic effect when used in combination with remdesivir which is a Western medicine used to treat COVID-19 (Tang et al., 2021). Moreover, the average dosage of these five medicinal materials in Group C revealed that the dosage of *P*. *australis* is lower than the standard dosage specified in the *Taiwan Herbal Pharmacopeia fourth Edition*. Group D consists of 8 medicinal materials, 7 of which are tonifying herbs (*Z*. *jujuba*, *L*. *chinense*, *H*. *polybotrys*, *C*. *pilosula*, *A*. *sinensis*, *P*. *quinquefolius*, and *P*. *ginseng*). Previous research has highlighted the significant role of tonifying medicinal materials in the COVID-19 pandemic, as they regulate immunity, helping to prevent immune storms that could harm the body, while also boosting immune function to enhance the body’s ability to fight viral infections (Sun et al., 2003; Yang et al., 2022). In addition, one study has shown that taking *P*. *ginseng* can enhance and maintain the effectiveness of COVID-19 vaccines, with particularly significant effects observed in individuals over the age of 50 (Yoon et al., 2023). Moreover, among the 8 medicinal materials in Group D, the dosage of *L*. *chinense* is higher than the standard dosage specified in the *Taiwan Herbal Pharmacopeia fourth Edition*. Furthermore, several of the 30 commonly used medicinal materials identified in this study have been reported to contain bioactive compounds with validated anti-COVID-19 activity. Notable examples include licoricesaponin A3 and glycyrrhetinic acid from *G*. *uralensis*; kuwanon C and mulberrofuran G from *M*. *alba*; quercetin and luteolin from *L*. *japonica* and *P*. *frutescens*; platycodin D from *P*. *grandiflorus*; baicalin and baicalein from *S*. *baicalensis*; ginsenoside compound K from *P*. *ginseng*; and obacunone, limonin, and nomilin from *C*. *reticulata* (Song et al., 2020; Kim et al., 2021; Liu et al., 2021; Magurano et al., 2021; Dissook et al., 2022; Kim et al., 2022a; Kim et al., 2022b; Yi et al., 2022; Boopathi et al., 2023; Gao et al., 2023). These compounds are primarily classified as terpenoids or polyphenols, indicating that future phytochemical screening for anti-COVID-19 candidates may be most fruitful when focused on these two classes of natural products.

In summary, the medicinal material combinations used in TCM pharmacies to combat COVID-19 can be categorized into four groups. Group A consists mainly of heat-clearing herbs, which are more suitable for moderate to severe COVID-19 patients to combat persistent high fever and inflammatory responses caused by viral infections. Group B is a mix of heat-clearing and tonifying herbs, making it suitable for mild COVID-19 patients as it combats the systemic inflammatory and boosts immunity simultaneously. Group C includes symptomatic treatments, which are appropriate for patients experiencing related symptoms such as cough and sputum production, or as an adjunct treatment in Group A and Group B therapies. Group D consists mainly of tonifying herbs, making it suitable for the public who have not yet contracted COVID-19, serving as an immunity-boosting regimen. Additionally, the dosages of the 30 commonly used medicinal materials vary across different pharmacies, and some of the average dosages do not conform to the standard dosages outlined in the *Taiwan Herbal Pharmacopeia fourth Edition*. This phenomenon reflects the unique approach TCM pharmacies take in selecting medicinal material dosages.

This study has several limitations. Firstly, the research is based on previous studies and makes pharmacological inferences regarding the commonly used anti-COVID-19 medicinal materials in TCM pharmacies. However, from a formulaic perspective, while the efficacy of Group A has been validated by research, Groups B-D still lack sufficient experimental evidence to support the effectiveness and safety of their formulae. Secondly, the doses of medicinal materials such as *C. cassia*, *I. tinctoria*, *P. australis*, and *L. chinense* mentioned in this study do not conform to the standard dosages outlined in the *Taiwan Herbal Pharmacopeia fourth Edition*, and thus their safety requires further research.

Despite these limitations, the results of this study not only provide numerous candidate medicinal materials and formulae for future anti-coronavirus treatments, but also contribute to the documentation of the ethnobotanical knowledge in combating COVID-19 from TCM pharmacy in Taiwan, thus supporting the achievement of the United Nations Sustainable Development Goals.

## 5 Conclusion

This study is the first ethnobotanical research on folk formulae for Anti-COVID-19 in Taiwan. It consolidates the use of 70 medicinal materials selected by TCM pharmacies for the prevention and treatment of COVID-19, with 30 commonly used medicinal materials categorized into four distinct formula groups, each targeting different stages of the disease. More importantly, this study employs an ethnobotanical research approach to not only identify potential herbs and formulae for combating COVID-19 but also to document and digitize traditional knowledge, ensuring its preservation in line with the United Nations Sustainable Development Goals. In the future., a “disease course-oriented specialized prescription model” will be adapted with reference to international literature and the development of COVID-19 symptoms reported by NIH. Different combinations of medicinal materials will be formulated in response to the rapid development of the disease, providing the clinical outcome with a reference for the selection of prescriptions for different stages of the disease.

## Data Availability

The original contributions presented in the study are included in the article/Supplementary Material, further inquiries can be directed to the corresponding authors.

## Glossary

ADR: *Angelica dahurica* (Hoffm.) Benth. and Hook.f. ex Franch. and Sav.
AFI: *Citrus × aurantium* L.
AGR: *Astragalus mongholicus* Bunge
ALI: *Alisma plantago-aquatica subsp. orientale* (Sam.) Sam.
AMR: *Atractylodes macrocephala* Koidz.
ARE: *Aster tataricus* L.f.
ARR: *Asarum heterotropoides* F.Schmidt
ASA: *Prunus sibirica* L.
ASE: *Eleutherococcus senticosus* (Rupr. and Maxim.) Maxim.
ASR: *Angelica sinensis* (Oliv.) Diels
ATH: *Agastache rugosa* (Fisch. and C.A.Mey.) Kuntze
BPR: *Bupleurum chinense* DC.
BTR: *Bletilla striata* (Thunb.) Rchb.f.
CLR: *Curcuma longa* L.
CMC: *Cinnamomum cassia* (L.) J.Presl **(Cortex)**
CMR: *Cinnamomum cassia* (L.) J.Presl **(Ramulus)**
COVID-19: Coronavirus Disease 2019
CRP: *Citrus reticulata* Blanco
CSF: *Chrysanthemum morifolium* Ramat.
CSR: *Codonopsis pilosula* (Franch.) Nannf.
CXR: *Ligusticum striatum* DC.
DSR: *Dioscorea oppositifolia* L.
EBF: *Eriobotrya japonica* (Thunb.) Lindl.
EDH: *Ephedra sinica* Stapf
EMC: *Eucommia ulmoides* Oliv.
FSR: *Forsythia suspensa* (Thunb.) Vahl
FTB: *Fritillaria thunbergii* Miq.
GLY: *Glycyrrhiza uralensis* Fisch.
GNR: *Glehnia littoralis* F.Schmidt ex Miq.
GSG: *Panax ginseng* C.A.Mey.
HCE: *Helminthostachys zeylanica* (L.) Hook.
HDR: *Hedysarum polybotrys* Hand.-Mazz.
HNH: *Houttuynia cordata* Thunb.
IPR: *Imperata cylindrica* (L.) Raeusch.
ISR: *Iris domestica* (L.) Goldblatt and Mabb.
ITR: *Isatis tinctoria* L.
JJB: *Ziziphus jujuba* Mill.
LBS: *Lilium lancifolium* Thunb.
LCF: *Lycium chinense* Mill.
LJF: *Lonicera japonica* Thunb.
LOP: *Lophatherum gracile* Brongn
LPH: *Lycopus lucidus* var. *hirtus* Regel
MNC: *Magnolia officinalis* Rehder and E.H.Wilson
MOHW: the Ministry of Health and Welfare
MRC: *Morus alba* L. **(Cortex)**
MRF: *Morus alba* L. **(Folium)**
MTH: *Mentha canadensis* L.
NBF: *Nelumbo nucifera* Gaertn.
NIH: National Institutes of Health
NPH: *Nepeta tenuifolia* Benth.
OPR: *Ophiopogon japonicus* (Thunb.) Ker Gawl.
PCR: *Platycodon grandiflorus* (Jacq.) A.DC.
PLC: *Perilla frutescens* (L.) Britton **(Caulis)**
PLF: *Perilla frutescens* (L.) Britton **(Folium)**
PLR: *Pinellia ternata* (Thunb.) Makino
PMR: *Phragmites australis* (Cav.) Trin. ex Steud.
POR: *Poria cocos* (Schwein.) F.A. Wolf
PQR: *Panax quinquefolius* L.
PRA: *Paeonia lactiflora* Pall.
PRR: *Pueraria montana* var. *chinensis* (Ohwi) Sanjappa and Pradeep
QFPDD: Qingfei Paidu Decoction
RFC: Relative frequency of citation
RMR: *Reynoutria multiflora* (Thunb.) Moldenke
RNR: *Rehmannia glutinosa* (Gaertn.) DC.
SCR: *Strobilanthes cusia* (Nees) Kuntze
SKE: *Saposhnikovia divaricata* (Turcz.) Schischk.
SME: *Salvia miltiorrhiza* Bunge
SNR: *Stemona sessilifolia* (Miq.) Miq.
SPR: *Scrophularia ningpoensis* Hemsl.
SPS: *Scaphium affine* (Mast.) Pierre
STF: *Siraitia grosvenorii* (Swingle) C.Jeffrey ex A.M.Lu and Zhi.Y.Zhang
STR: *Scutellaria baicalensis* Georgi
TCM: traditional Chinese medicine
TSF: *Trichosanthes kirilowii* Maxim.
TWD97: the Taiwan Geodetic Datum 1997
TXH: *Taraxacum mongolicum* Hand.-Mazz.
WHO: World Health Organization
ZGB: *Zingiber officinale* Roscoe
