# Supplementary material for: Ethnobotanical survey of herbal medicines for anti-COVID-19 used by traditional Chinese medicine pharmacies in Taiwan
Source: Front Pharmacol. 2025 Sep 3;16:1586334. doi: 10.3389/fphar.2025.1586334 (PMC12440865; doi:10.3389/fphar.2025.1586334)
Supplement: Supplementary file 1 [file DataSheet1.docx]

**Supplementary Data**

| **Table S1. Sampling of Counties and Cities in Taiwan** | | | | |  |
| --- | --- | --- | --- | --- | --- |
| Region | Code | Total Number of TCM Pharmacies | Expected Number of TCM Pharmacies Visited**^a^** | Actual Number of TCM Pharmacies Visited | |
| Keelung City | KEL | 77 | 1 | 1 | |
| Taipei City | TPE | 578 | 7 | 8 | |
| New Taipei City | NTPC | 880 | 11 | 9 | |
| Taoyuan City | TYN | 509 | 6 | 5 | |
| Hsinchu County | HSZ | 238 | 3 | 2 | |
| Miaoli County | ZMI | 166 | 2 | 1 | |
| Taichung City | TXG | 1039 | 12 | 17 | |
| Changhua County | CHW | 687 | 8 | 7 | |
| Nantou County | NTC | 377 | 5 | 4 | |
| Yunlin County | YUN | 334 | 4 | 3 | |
| Chiayi County | CYI | 427 | 5 | 4 | |
| Tainan City | TNN | 1000 | 12 | 12 | |
| Kaohsiung City | KHH | 1350 | 16 | 22 | |
| Pingtung County | PIF | 403 | 5 | 6 | |
| Yilan County | ILA | 157 | 2 | 2 | |
| Hualien County | HUN | 78 | 1 | 2 | |
| Taitung County | TTT | 48 | 1 | 1 | |
| Total |  | 8348 | 101 | 106 | |

TCM: Traditional Chinese Medicine

^a^ The expected number of TCM pharmacies visited is the number of TCM pharmacies in each region divided by the total number of TCM pharmacies multiplied by 100 (rounded).

| **Table S2. Frequency Contingency Table of 30 Commonly used Medicinal Materials in 61 formulae** | | | | | | | | | | | | | | | | | | | | | | | | | | | | | | | | | | | | | | | | | | | | | | | | | | | | | | | | | | | | | |
| --- | --- | --- | --- | --- | --- | --- | --- | --- | --- | --- | --- | --- | --- | --- | --- | --- | --- | --- | --- | --- | --- | --- | --- | --- | --- | --- | --- | --- | --- | --- | --- | --- | --- | --- | --- | --- | --- | --- | --- | --- | --- | --- | --- | --- | --- | --- | --- | --- | --- | --- | --- | --- | --- | --- | --- | --- | --- | --- | --- | --- | --- |
|  |  | **GLY** | | **HNH** | | **JJB** | | **MTH** | | **LCF** | | **AGR** | | **HDR** | | **MRF** | | **LJF** | | **OPR** | | **SKE** | | **CMR** | | **PCR** | | **NPH** | | **PLF** | | **CSF** | | **CSR** | | **AMR** | | **ASR** | | **STR** | | **PQR** | | **ZGB** | | **TSF** | | **ITR** | | **CXR** | | **ATH** | | **PMR** | | **GSG** | | **SCR** | | **CRP** | |
|  |  | **Y** | **N** | **Y** | **N** | **Y** | **N** | **Y** | **N** | **Y** | **N** | **Y** | **N** | **Y** | **N** | **Y** | **N** | **Y** | **N** | **Y** | **N** | **Y** | **N** | **Y** | **N** | **Y** | **N** | **Y** | **N** | **Y** | **N** | **Y** | **N** | **Y** | **N** | **Y** | **N** | **Y** | **N** | **Y** | **N** | **Y** | **N** | **Y** | **N** | **Y** | **N** | **Y** | **N** | **Y** | **N** | **Y** | **N** | **Y** | **N** | **Y** | **N** | **Y** | **N** | **Y** | **N** |
| **GLY** | **Y** | **35** | **0** |  |  |  |  |  |  |  |  |  |  |  |  |  |  |  |  |  |  |  |  |  |  |  |  |  |  |  |  |  |  |  |  |  |  |  |  |  |  |  |  |  |  |  |  |  |  |  |  |  |  |  |  |  |  |  |  |  |  |
|  | **N** | **0** | **35** |  |  |  |  |  |  |  |  |  |  |  |  |  |  |  |  |  |  |  |  |  |  |  |  |  |  |  |  |  |  |  |  |  |  |  |  |  |  |  |  |  |  |  |  |  |  |  |  |  |  |  |  |  |  |  |  |  |  |
| **HNH** | **Y** | **24** | **5** | **29** | **0** |  |  |  |  |  |  |  |  |  |  |  |  |  |  |  |  |  |  |  |  |  |  |  |  |  |  |  |  |  |  |  |  |  |  |  |  |  |  |  |  |  |  |  |  |  |  |  |  |  |  |  |  |  |  |  |  |
|  | **N** | **11** | **21** | **0** | **29** |  |  |  |  |  |  |  |  |  |  |  |  |  |  |  |  |  |  |  |  |  |  |  |  |  |  |  |  |  |  |  |  |  |  |  |  |  |  |  |  |  |  |  |  |  |  |  |  |  |  |  |  |  |  |  |  |
| **JJB** | **Y** | **10** | **18** | **10** | **18** | **28** | **0** |  |  |  |  |  |  |  |  |  |  |  |  |  |  |  |  |  |  |  |  |  |  |  |  |  |  |  |  |  |  |  |  |  |  |  |  |  |  |  |  |  |  |  |  |  |  |  |  |  |  |  |  |  |  |
|  | **N** | **25** | **8** | **19** | **14** | **0** | **28** |  |  |  |  |  |  |  |  |  |  |  |  |  |  |  |  |  |  |  |  |  |  |  |  |  |  |  |  |  |  |  |  |  |  |  |  |  |  |  |  |  |  |  |  |  |  |  |  |  |  |  |  |  |  |
| **MTH** | **Y** | **18** | **4** | **18** | **4** | **7** | **15** | **22** | **0** |  |  |  |  |  |  |  |  |  |  |  |  |  |  |  |  |  |  |  |  |  |  |  |  |  |  |  |  |  |  |  |  |  |  |  |  |  |  |  |  |  |  |  |  |  |  |  |  |  |  |  |  |
|  | **N** | **17** | **22** | **11** | **28** | **21** | **18** | **0** | **22** |  |  |  |  |  |  |  |  |  |  |  |  |  |  |  |  |  |  |  |  |  |  |  |  |  |  |  |  |  |  |  |  |  |  |  |  |  |  |  |  |  |  |  |  |  |  |  |  |  |  |  |  |
| **LCF** | **Y** | **4** | **18** | **2** | **20** | **17** | **5** | **0** | **22** | **22** | **0** |  |  |  |  |  |  |  |  |  |  |  |  |  |  |  |  |  |  |  |  |  |  |  |  |  |  |  |  |  |  |  |  |  |  |  |  |  |  |  |  |  |  |  |  |  |  |  |  |  |  |
|  | **N** | **31** | **8** | **27** | **12** | **11** | **28** | **22** | **17** | **0** | **22** |  |  |  |  |  |  |  |  |  |  |  |  |  |  |  |  |  |  |  |  |  |  |  |  |  |  |  |  |  |  |  |  |  |  |  |  |  |  |  |  |  |  |  |  |  |  |  |  |  |  |
| **AGR** | **Y** | **12** | **8** | **11** | **9** | **12** | **8** | **9** | **11** | **6** | **14** | **20** | **0** |  |  |  |  |  |  |  |  |  |  |  |  |  |  |  |  |  |  |  |  |  |  |  |  |  |  |  |  |  |  |  |  |  |  |  |  |  |  |  |  |  |  |  |  |  |  |  |  |
|  | **N** | **23** | **18** | **18** | **23** | **16** | **25** | **13** | **28** | **16** | **28** | **0** | **20** |  |  |  |  |  |  |  |  |  |  |  |  |  |  |  |  |  |  |  |  |  |  |  |  |  |  |  |  |  |  |  |  |  |  |  |  |  |  |  |  |  |  |  |  |  |  |  |  |
| **HDR** | **Y** | **6** | **13** | **6** | **13** | **16** | **3** | **5** | **14** | **13** | **6** | **0** | **19** | **19** | **0** |  |  |  |  |  |  |  |  |  |  |  |  |  |  |  |  |  |  |  |  |  |  |  |  |  |  |  |  |  |  |  |  |  |  |  |  |  |  |  |  |  |  |  |  |  |  |
|  | **N** | **29** | **13** | **23** | **19** | **12** | **30** | **17** | **25** | **9** | **25** | **20** | **22** | **0** | **19** |  |  |  |  |  |  |  |  |  |  |  |  |  |  |  |  |  |  |  |  |  |  |  |  |  |  |  |  |  |  |  |  |  |  |  |  |  |  |  |  |  |  |  |  |  |  |
| **MRF** | **Y** | **16** | **1** | **17** | **0** | **8** | **9** | **13** | **4** | **2** | **15** | **7** | **10** | **5** | **12** | **17** | **0** |  |  |  |  |  |  |  |  |  |  |  |  |  |  |  |  |  |  |  |  |  |  |  |  |  |  |  |  |  |  |  |  |  |  |  |  |  |  |  |  |  |  |  |  |
|  | **N** | **19** | **25** | **12** | **32** | **20** | **24** | **9** | **35** | **20** | **35** | **13** | **31** | **14** | **30** | **0** | **17** |  |  |  |  |  |  |  |  |  |  |  |  |  |  |  |  |  |  |  |  |  |  |  |  |  |  |  |  |  |  |  |  |  |  |  |  |  |  |  |  |  |  |  |  |
| **LJF** | **Y** | **10** | **4** | **5** | **9** | **1** | **13** | **6** | **8** | **3** | **11** | **5** | **9** | **2** | **12** | **2** | **12** | **14** | **0** |  |  |  |  |  |  |  |  |  |  |  |  |  |  |  |  |  |  |  |  |  |  |  |  |  |  |  |  |  |  |  |  |  |  |  |  |  |  |  |  |  |  |
|  | **N** | **25** | **22** | **24** | **23** | **27** | **20** | **16** | **31** | **19** | **31** | **15** | **32** | **17** | **30** | **15** | **32** | **0** | **14** |  |  |  |  |  |  |  |  |  |  |  |  |  |  |  |  |  |  |  |  |  |  |  |  |  |  |  |  |  |  |  |  |  |  |  |  |  |  |  |  |  |  |
| **OPR** | **Y** | **8** | **4** | **7** | **5** | **3** | **9** | **3** | **9** | **3** | **9** | **2** | **10** | **2** | **10** | **2** | **10** | **1** | **11** | **12** | **0** |  |  |  |  |  |  |  |  |  |  |  |  |  |  |  |  |  |  |  |  |  |  |  |  |  |  |  |  |  |  |  |  |  |  |  |  |  |  |  |  |
|  | **N** | **27** | **22** | **22** | **27** | **25** | **24** | **19** | **30** | **19** | **30** | **18** | **31** | **17** | **32** | **15** | **34** | **13** | **36** | **0** | **12** |  |  |  |  |  |  |  |  |  |  |  |  |  |  |  |  |  |  |  |  |  |  |  |  |  |  |  |  |  |  |  |  |  |  |  |  |  |  |  |  |
| **SKE** | **Y** | **9** | **2** | **9** | **2** | **2** | **9** | **7** | **4** | **0** | **11** | **5** | **6** | **1** | **10** | **5** | **6** | **4** | **7** | **2** | **9** | **11** | **0** |  |  |  |  |  |  |  |  |  |  |  |  |  |  |  |  |  |  |  |  |  |  |  |  |  |  |  |  |  |  |  |  |  |  |  |  |  |  |
|  | **N** | **26** | **24** | **20** | **30** | **26** | **24** | **15** | **35** | **22** | **35** | **15** | **35** | **18** | **32** | **12** | **38** | **10** | **40** | **10** | **40** | **0** | **11** |  |  |  |  |  |  |  |  |  |  |  |  |  |  |  |  |  |  |  |  |  |  |  |  |  |  |  |  |  |  |  |  |  |  |  |  |  |  |
| **CMR** | **Y** | **7** | **4** | **9** | **2** | **8** | **3** | **8** | **3** | **1** | **10** | **5** | **6** | **4** | **7** | **7** | **4** | **1** | **10** | **0** | **11** | **2** | **9** | **11** | **0** |  |  |  |  |  |  |  |  |  |  |  |  |  |  |  |  |  |  |  |  |  |  |  |  |  |  |  |  |  |  |  |  |  |  |  |  |
|  | **N** | **28** | **22** | **20** | **30** | **20** | **30** | **14** | **36** | **21** | **36** | **15** | **35** | **15** | **35** | **10** | **40** | **13** | **37** | **12** | **38** | **9** | **41** | **0** | **11** |  |  |  |  |  |  |  |  |  |  |  |  |  |  |  |  |  |  |  |  |  |  |  |  |  |  |  |  |  |  |  |  |  |  |  |  |
| **PCR** | **Y** | **10** | **1** | **9** | **2** | **0** | **11** | **6** | **5** | **0** | **11** | **3** | **8** | **0** | **11** | **2** | **9** | **4** | **7** | **6** | **5** | **4** | **7** | **1** | **10** | **11** | **0** |  |  |  |  |  |  |  |  |  |  |  |  |  |  |  |  |  |  |  |  |  |  |  |  |  |  |  |  |  |  |  |  |  |  |
|  | **N** | **25** | **25** | **20** | **30** | **28** | **22** | **16** | **34** | **22** | **34** | **17** | **33** | **19** | **31** | **15** | **35** | **10** | **40** | **6** | **44** | **7** | **43** | **10** | **40** | **0** | **11** |  |  |  |  |  |  |  |  |  |  |  |  |  |  |  |  |  |  |  |  |  |  |  |  |  |  |  |  |  |  |  |  |  |  |
| **NPH** | **Y** | **9** | **1** | **8** | **2** | **2** | **8** | **9** | **1** | **0** | **10** | **3** | **7** | **2** | **8** | **6** | **4** | **4** | **6** | **1** | **9** | **7** | **3** | **2** | **8** | **3** | **7** | **10** | **0** |  |  |  |  |  |  |  |  |  |  |  |  |  |  |  |  |  |  |  |  |  |  |  |  |  |  |  |  |  |  |  |  |
|  | **N** | **26** | **25** | **21** | **30** | **26** | **25** | **13** | **38** | **22** | **38** | **17** | **34** | **17** | **34** | **11** | **40** | **10** | **41** | **11** | **40** | **4** | **47** | **9** | **42** | **8** | **43** | **0** | **10** |  |  |  |  |  |  |  |  |  |  |  |  |  |  |  |  |  |  |  |  |  |  |  |  |  |  |  |  |  |  |  |  |
| **PLF** | **Y** | **8** | **1** | **7** | **2** | **0** | **9** | **4** | **5** | **1** | **8** | **4** | **5** | **0** | **9** | **2** | **7** | **4** | **5** | **4** | **5** | **2** | **7** | **0** | **9** | **6** | **3** | **2** | **7** | **9** | **0** |  |  |  |  |  |  |  |  |  |  |  |  |  |  |  |  |  |  |  |  |  |  |  |  |  |  |  |  |  |  |
|  | **N** | **27** | **25** | **22** | **30** | **28** | **24** | **18** | **34** | **21** | **34** | **16** | **36** | **19** | **33** | **15** | **37** | **10** | **42** | **8** | **44** | **9** | **43** | **11** | **41** | **5** | **47** | **8** | **44** | **0** | **9** |  |  |  |  |  |  |  |  |  |  |  |  |  |  |  |  |  |  |  |  |  |  |  |  |  |  |  |  |  |  |
| **CSF** | **Y** | **6** | **2** | **6** | **2** | **1** | **7** | **1** | **7** | **2** | **6** | **1** | **7** | **1** | **7** | **1** | **7** | **3** | **5** | **5** | **3** | **0** | **8** | **0** | **8** | **4** | **4** | **0** | **8** | **3** | **5** | **8** | **0** |  |  |  |  |  |  |  |  |  |  |  |  |  |  |  |  |  |  |  |  |  |  |  |  |  |  |  |  |
|  | **N** | **29** | **24** | **23** | **30** | **27** | **26** | **21** | **32** | **20** | **32** | **19** | **34** | **18** | **35** | **16** | **37** | **11** | **42** | **7** | **46** | **11** | **42** | **11** | **42** | **7** | **46** | **10** | **43** | **6** | **47** | **0** | **8** |  |  |  |  |  |  |  |  |  |  |  |  |  |  |  |  |  |  |  |  |  |  |  |  |  |  |  |  |
| **CSR** | **Y** | **2** | **6** | **2** | **6** | **6** | **2** | **1** | **7** | **6** | **2** | **5** | **3** | **3** | **5** | **0** | **8** | **2** | **6** | **2** | **6** | **0** | **8** | **1** | **7** | **1** | **7** | **0** | **8** | **2** | **6** | **0** | **8** | **8** | **0** |  |  |  |  |  |  |  |  |  |  |  |  |  |  |  |  |  |  |  |  |  |  |  |  |  |  |
|  | **N** | **33** | **20** | **27** | **26** | **22** | **31** | **21** | **32** | **16** | **32** | **15** | **38** | **16** | **37** | **17** | **36** | **12** | **41** | **10** | **43** | **11** | **42** | **10** | **43** | **10** | **43** | **10** | **43** | **7** | **46** | **8** | **45** | **0** | **8** |  |  |  |  |  |  |  |  |  |  |  |  |  |  |  |  |  |  |  |  |  |  |  |  |  |  |
| **AMR** | **Y** | **6** | **1** | **3** | **4** | **1** | **6** | **2** | **5** | **0** | **7** | **4** | **3** | **0** | **7** | **1** | **6** | **3** | **4** | **0** | **7** | **4** | **3** | **2** | **5** | **2** | **5** | **3** | **4** | **3** | **4** | **0** | **7** | **1** | **6** | **7** | **0** |  |  |  |  |  |  |  |  |  |  |  |  |  |  |  |  |  |  |  |  |  |  |  |  |
|  | **N** | **29** | **25** | **26** | **28** | **27** | **27** | **20** | **34** | **22** | **34** | **16** | **38** | **19** | **35** | **16** | **38** | **11** | **43** | **12** | **42** | **7** | **47** | **9** | **45** | **9** | **45** | **7** | **47** | **6** | **48** | **8** | **46** | **7** | **47** | **0** | **7** |  |  |  |  |  |  |  |  |  |  |  |  |  |  |  |  |  |  |  |  |  |  |  |  |
| **ASR** | **Y** | **2** | **5** | **1** | **6** | **6** | **1** | **0** | **7** | **6** | **1** | **0** | **7** | **6** | **1** | **0** | **7** | **0** | **7** | **2** | **5** | **1** | **6** | **1** | **6** | **1** | **6** | **0** | **7** | **0** | **7** | **0** | **7** | **2** | **5** | **0** | **7** | **7** | **0** |  |  |  |  |  |  |  |  |  |  |  |  |  |  |  |  |  |  |  |  |  |  |
|  | **N** | **33** | **21** | **28** | **26** | **22** | **32** | **22** | **32** | **16** | **32** | **20** | **34** | **13** | **41** | **17** | **37** | **14** | **40** | **10** | **44** | **10** | **44** | **10** | **44** | **10** | **44** | **10** | **44** | **9** | **45** | **8** | **46** | **6** | **48** | **7** | **47** | **0** | **7** |  |  |  |  |  |  |  |  |  |  |  |  |  |  |  |  |  |  |  |  |  |  |
| **STR** | **Y** | **6** | **0** | **5** | **1** | **0** | **6** | **5** | **1** | **0** | **6** | **1** | **5** | **1** | **5** | **4** | **2** | **1** | **5** | **1** | **5** | **3** | **3** | **1** | **5** | **1** | **5** | **5** | **1** | **0** | **6** | **0** | **6** | **0** | **6** | **1** | **5** | **0** | **6** | **6** | **0** |  |  |  |  |  |  |  |  |  |  |  |  |  |  |  |  |  |  |  |  |
|  | **N** | **29** | **26** | **24** | **31** | **28** | **27** | **17** | **38** | **22** | **38** | **19** | **36** | **18** | **37** | **13** | **42** | **13** | **42** | **11** | **44** | **8** | **47** | **10** | **45** | **10** | **45** | **5** | **50** | **9** | **46** | **8** | **47** | **8** | **47** | **6** | **49** | **7** | **48** | **0** | **6** |  |  |  |  |  |  |  |  |  |  |  |  |  |  |  |  |  |  |  |  |
| **PQR** | **Y** | **2** | **4** | **0** | **6** | **4** | **2** | **0** | **6** | **5** | **1** | **1** | **5** | **3** | **3** | **0** | **6** | **0** | **6** | **2** | **4** | **0** | **6** | **0** | **6** | **0** | **6** | **0** | **6** | **0** | **6** | **0** | **6** | **0** | **6** | **0** | **6** | **1** | **5** | **0** | **6** | **6** | **0** |  |  |  |  |  |  |  |  |  |  |  |  |  |  |  |  |  |  |
|  | **N** | **33** | **22** | **29** | **26** | **24** | **31** | **22** | **33** | **17** | **33** | **19** | **36** | **16** | **39** | **17** | **38** | **14** | **41** | **10** | **45** | **11** | **44** | **11** | **44** | **11** | **44** | **10** | **45** | **9** | **46** | **8** | **47** | **8** | **47** | **7** | **48** | **6** | **49** | **6** | **49** | **0** | **6** |  |  |  |  |  |  |  |  |  |  |  |  |  |  |  |  |  |  |
| **ZGB** | **Y** | **5** | **1** | **5** | **1** | **5** | **1** | **4** | **2** | **0** | **6** | **4** | **2** | **1** | **5** | **4** | **2** | **0** | **6** | **0** | **6** | **1** | **5** | **6** | **0** | **0** | **6** | **1** | **5** | **0** | **6** | **0** | **6** | **0** | **6** | **2** | **4** | **0** | **6** | **1** | **5** | **0** | **6** | **6** | **0** |  |  |  |  |  |  |  |  |  |  |  |  |  |  |  |  |
|  | **N** | **30** | **25** | **24** | **31** | **23** | **32** | **18** | **37** | **22** | **37** | **16** | **39** | **18** | **37** | **13** | **42** | **14** | **41** | **12** | **43** | **10** | **45** | **5** | **50** | **11** | **44** | **9** | **46** | **9** | **46** | **8** | **47** | **8** | **47** | **5** | **50** | **7** | **48** | **5** | **50** | **6** | **49** | **0** | **6** |  |  |  |  |  |  |  |  |  |  |  |  |  |  |  |  |
| **TSF** | **Y** | **5** | **0** | **5** | **0** | **0** | **5** | **5** | **0** | **0** | **5** | **1** | **4** | **1** | **4** | **4** | **1** | **1** | **4** | **1** | **4** | **3** | **2** | **0** | **5** | **1** | **4** | **5** | **0** | **0** | **5** | **0** | **5** | **0** | **5** | **0** | **5** | **0** | **5** | **5** | **0** | **0** | **5** | **0** | **5** | **5** | **0** |  |  |  |  |  |  |  |  |  |  |  |  |  |  |
|  | **N** | **30** | **26** | **24** | **32** | **28** | **28** | **17** | **39** | **22** | **39** | **19** | **37** | **18** | **38** | **13** | **43** | **13** | **43** | **11** | **45** | **8** | **48** | **11** | **45** | **10** | **46** | **5** | **51** | **9** | **47** | **8** | **48** | **8** | **48** | **7** | **49** | **7** | **49** | **1** | **55** | **6** | **50** | **6** | **50** | **0** | **5** |  |  |  |  |  |  |  |  |  |  |  |  |  |  |
| **ITR** | **Y** | **3** | **2** | **3** | **2** | **1** | **4** | **2** | **3** | **0** | **5** | **1** | **4** | **1** | **4** | **1** | **4** | **3** | **2** | **0** | **5** | **1** | **4** | **0** | **5** | **0** | **5** | **1** | **4** | **1** | **4** | **0** | **5** | **1** | **4** | **1** | **4** | **0** | **5** | **0** | **5** | **0** | **5** | **0** | **5** | **0** | **5** | **5** | **0** |  |  |  |  |  |  |  |  |  |  |  |  |
|  | **N** | **32** | **24** | **26** | **30** | **27** | **29** | **20** | **36** | **22** | **36** | **19** | **37** | **18** | **38** | **16** | **40** | **11** | **45** | **12** | **44** | **10** | **46** | **11** | **45** | **11** | **45** | **9** | **47** | **8** | **48** | **8** | **48** | **7** | **49** | **6** | **50** | **7** | **49** | **6** | **50** | **6** | **50** | **6** | **50** | **5** | **51** | **0** | **5** |  |  |  |  |  |  |  |  |  |  |  |  |
| **CXR** | **Y** | **2** | **3** | **0** | **5** | **4** | **1** | **0** | **5** | **4** | **1** | **0** | **5** | **4** | **1** | **0** | **5** | **0** | **5** | **1** | **4** | **0** | **5** | **1** | **4** | **1** | **4** | **0** | **5** | **0** | **5** | **0** | **5** | **2** | **3** | **1** | **4** | **4** | **1** | **0** | **5** | **0** | **5** | **0** | **5** | **0** | **5** | **0** | **5** | **5** | **0** |  |  |  |  |  |  |  |  |  |  |
|  | **N** | **33** | **23** | **29** | **27** | **24** | **32** | **22** | **34** | **18** | **34** | **20** | **36** | **15** | **41** | **17** | **39** | **14** | **42** | **11** | **45** | **11** | **45** | **10** | **46** | **10** | **46** | **10** | **46** | **9** | **47** | **8** | **48** | **6** | **50** | **6** | **50** | **3** | **53** | **6** | **50** | **6** | **50** | **6** | **50** | **5** | **51** | **5** | **51** | **0** | **5** |  |  |  |  |  |  |  |  |  |  |
| **ATH** | **Y** | **3** | **2** | **4** | **1** | **2** | **3** | **3** | **2** | **0** | **5** | **3** | **2** | **1** | **4** | **0** | **5** | **3** | **2** | **0** | **5** | **2** | **3** | **2** | **3** | **2** | **3** | **2** | **3** | **3** | **2** | **0** | **5** | **2** | **3** | **3** | **2** | **0** | **5** | **0** | **5** | **0** | **5** | **1** | **4** | **0** | **5** | **1** | **4** | **0** | **5** | **5** | **0** |  |  |  |  |  |  |  |  |
|  | **N** | **32** | **24** | **25** | **31** | **26** | **30** | **19** | **37** | **22** | **37** | **17** | **39** | **18** | **38** | **17** | **39** | **11** | **45** | **12** | **44** | **9** | **47** | **9** | **47** | **9** | **47** | **8** | **48** | **6** | **50** | **8** | **48** | **6** | **50** | **4** | **52** | **7** | **49** | **6** | **50** | **6** | **50** | **5** | **51** | **5** | **51** | **4** | **52** | **5** | **51** | **0** | **5** |  |  |  |  |  |  |  |  |
| **PMR** | **Y** | **4** | **1** | **5** | **0** | **1** | **4** | **1** | **4** | **0** | **5** | **1** | **4** | **0** | **5** | **1** | **4** | **0** | **5** | **4** | **1** | **1** | **4** | **0** | **5** | **4** | **1** | **0** | **5** | **3** | **2** | **4** | **1** | **0** | **5** | **0** | **5** | **0** | **5** | **0** | **5** | **0** | **5** | **0** | **5** | **0** | **5** | **0** | **5** | **0** | **5** | **0** | **5** | **5** | **0** |  |  |  |  |  |  |
|  | **N** | **31** | **25** | **24** | **32** | **27** | **29** | **21** | **35** | **22** | **35** | **19** | **37** | **19** | **37** | **16** | **40** | **14** | **42** | **8** | **48** | **10** | **46** | **11** | **45** | **7** | **49** | **10** | **46** | **6** | **50** | **4** | **52** | **8** | **48** | **7** | **49** | **7** | **49** | **6** | **50** | **6** | **50** | **6** | **50** | **5** | **51** | **5** | **51** | **5** | **51** | **5** | **51** | **0** | **5** |  |  |  |  |  |  |
| **GSG** | **Y** | **2** | **3** | **0** | **5** | **3** | **2** | **0** | **5** | **4** | **1** | **1** | **4** | **2** | **3** | **0** | **5** | **0** | **5** | **1** | **4** | **0** | **5** | **0** | **5** | **0** | **5** | **0** | **5** | **0** | **5** | **0** | **5** | **0** | **5** | **0** | **5** | **2** | **3** | **0** | **5** | **2** | **3** | **0** | **5** | **0** | **5** | **0** | **5** | **2** | **3** | **0** | **5** | **0** | **5** | **5** | **0** |  |  |  |  |
|  | **N** | **33** | **23** | **29** | **27** | **25** | **31** | **22** | **34** | **18** | **34** | **19** | **37** | **17** | **39** | **17** | **39** | **14** | **42** | **11** | **45** | **11** | **45** | **11** | **45** | **11** | **45** | **10** | **46** | **9** | **47** | **8** | **48** | **8** | **48** | **7** | **49** | **5** | **51** | **6** | **50** | **4** | **52** | **6** | **50** | **5** | **51** | **5** | **51** | **3** | **53** | **5** | **51** | **5** | **51** | **0** | **5** |  |  |  |  |
| **SCR** | **Y** | **4** | **0** | **3** | **1** | **0** | **4** | **2** | **2** | **0** | **4** | **1** | **3** | **1** | **3** | **1** | **3** | **2** | **2** | **1** | **3** | **3** | **1** | **0** | **4** | **2** | **2** | **2** | **2** | **0** | **4** | **0** | **4** | **0** | **4** | **0** | **4** | **1** | **3** | **2** | **2** | **0** | **4** | **0** | **4** | **2** | **2** | **0** | **4** | **0** | **4** | **0** | **4** | **0** | **4** | **0** | **4** | **4** | **0** |  |  |
|  | **N** | **31** | **26** | **26** | **31** | **28** | **29** | **20** | **37** | **22** | **37** | **19** | **38** | **18** | **39** | **16** | **41** | **12** | **45** | **11** | **46** | **8** | **49** | **11** | **46** | **9** | **48** | **8** | **49** | **9** | **48** | **8** | **49** | **8** | **49** | **7** | **50** | **6** | **51** | **4** | **53** | **6** | **51** | **6** | **51** | **3** | **54** | **5** | **52** | **5** | **52** | **5** | **52** | **5** | **52** | **5** | **52** | **0** | **4** |  |  |
| **CRP** | **Y** | **4** | **0** | **3** | **1** | **1** | **3** | **2** | **2** | **1** | **3** | **1** | **3** | **1** | **3** | **2** | **2** | **3** | **1** | **0** | **4** | **1** | **3** | **2** | **2** | **2** | **2** | **1** | **3** | **1** | **3** | **1** | **3** | **1** | **3** | **1** | **3** | **0** | **4** | **1** | **3** | **0** | **4** | **1** | **3** | **0** | **4** | **0** | **4** | **0** | **4** | **1** | **3** | **0** | **4** | **0** | **4** | **0** | **4** | **4** | **0** |
|  | **N** | **31** | **26** | **26** | **31** | **27** | **30** | **20** | **37** | **21** | **37** | **19** | **38** | **18** | **39** | **15** | **42** | **11** | **46** | **12** | **45** | **10** | **47** | **9** | **48** | **9** | **48** | **9** | **48** | **8** | **49** | **7** | **50** | **7** | **50** | **6** | **51** | **7** | **50** | **5** | **52** | **6** | **51** | **5** | **52** | **5** | **52** | **5** | **52** | **5** | **52** | **4** | **53** | **5** | **52** | **5** | **52** | **4** | **53** | **0** | **4** |

**Gly, *Glycyrrhiza uralensis* Fisch.; HNH, *Houttuynia cordata* Thunb.; JJB, *Ziziphus jujuba* Mill.; MTH, *Mentha canadensis* L.; LCF, *Lycium chinense* Mill.; AGR, *Astragalus mongholicus* Bunge; HDR, *Hedysarum polybotrys* Hand.-Mazz.; MRF, *Morus alba* L.; LJF, *Lonicera japonica* Thunb.; OPR, *Ophiopogon japonicus* (Thunb.) Ker Gawl.; SKE, *Saposhnikovia divaricata* (Turcz.) Schischk.; CMR, *Cinnamomum cassia* (L.) J.Presl; PCR, *Platycodon grandiflorus* (Jacq.) A.DC.; NPH, *Nepeta tenuifolia* Benth.; PLF, *Perilla frutescens* (L.) Britton; CSF, *Chrysanthemum morifolium* Ramat.; CSR, *Codonopsis pilosula* (Franch.) Nannf.; AMR, *Atractylodes macrocephala* Koidz.; ASR, *Angelica sinensis* (Oliv.) Diels; STR, *Scutellaria baicalensis* Georgi; PQR, *Panax quinquefolius* L.; ZGB, *Zingiber officinale* Roscoe; TSF, *Trichosanthes kirilowii* Maxim.; ITR, *Isatis tinctoria* L.; CXR, *Ligusticum striatum* DC.; ATH, *Agastache rugosa* (Fisch. & C.A.Mey.) Kuntze; PMR, *Phragmites australis* (Cav.) Trin. ex Steud.; GSG, *Panax ginseng* C.A.Mey.; SCR, *Strobilanthes cusia* (Nees) Kuntze; CRP, *Citrus reticulata* Blanco**

| 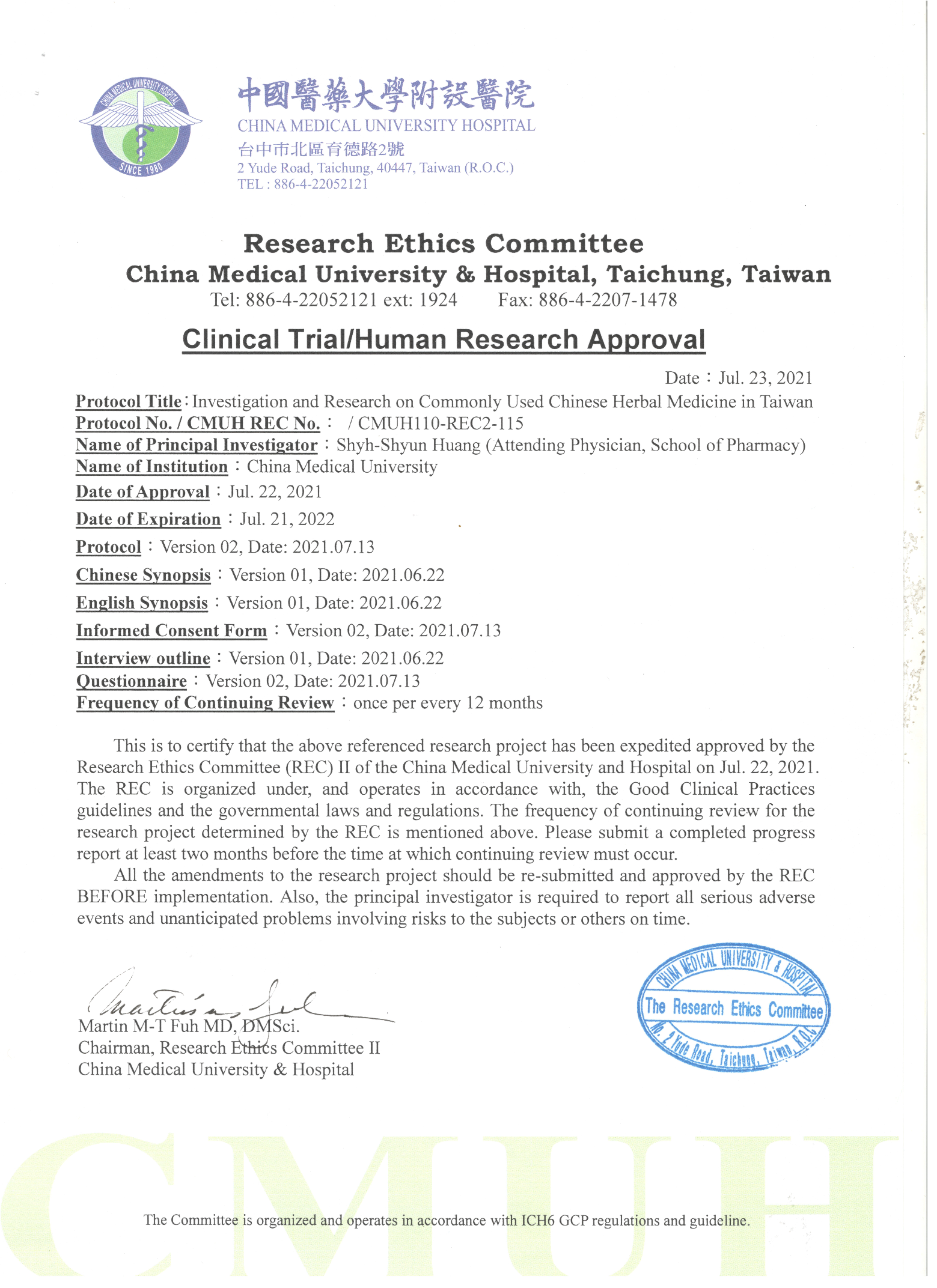 |
| --- |
| **Figure S1. Human Research/Human Research Approval Certificate**  **(No. CMUH110-REC2-115)** |

| 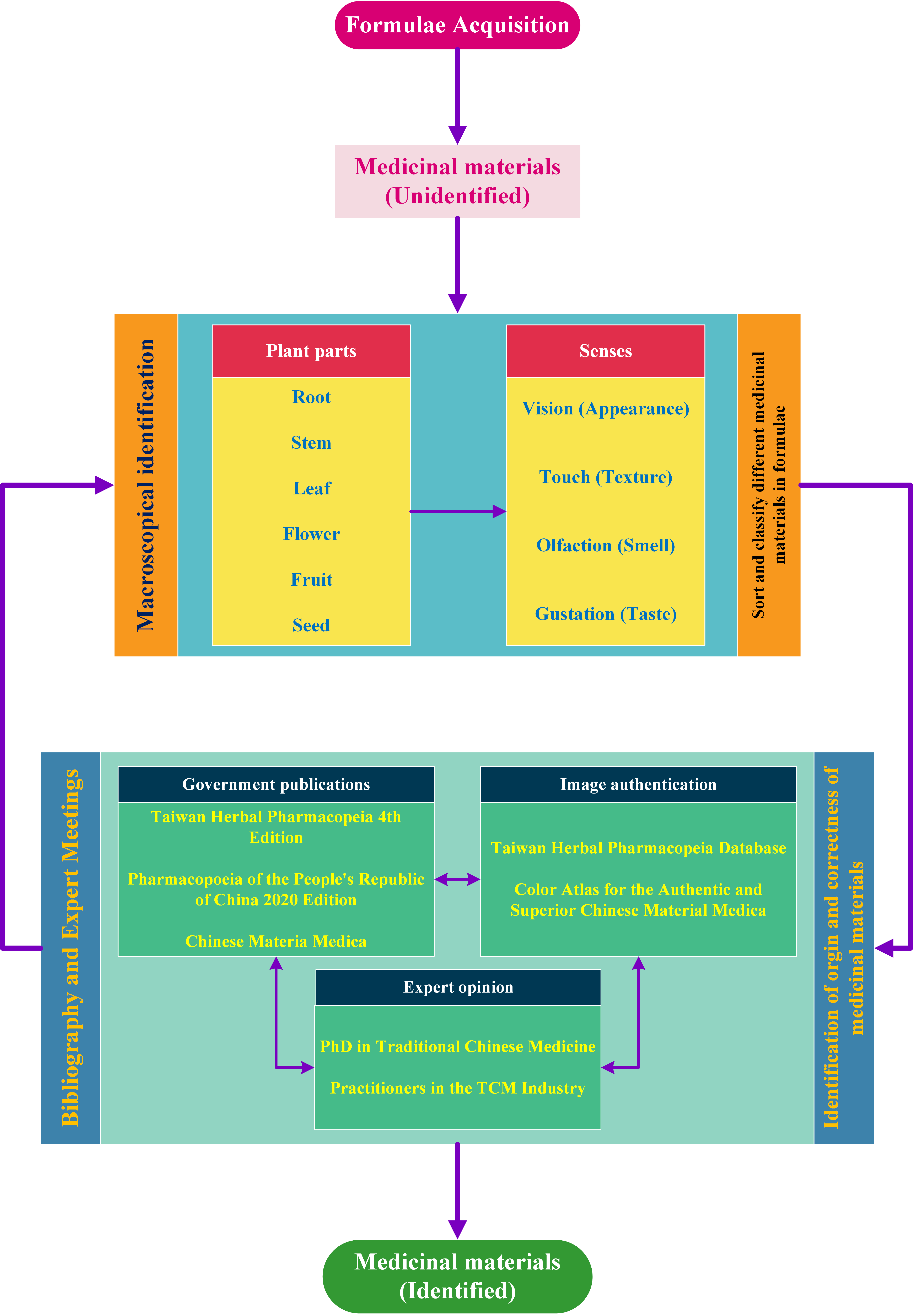 |
| --- |
| **Figure S2. Flow Chart of Medicinal Material Authentication** |

| 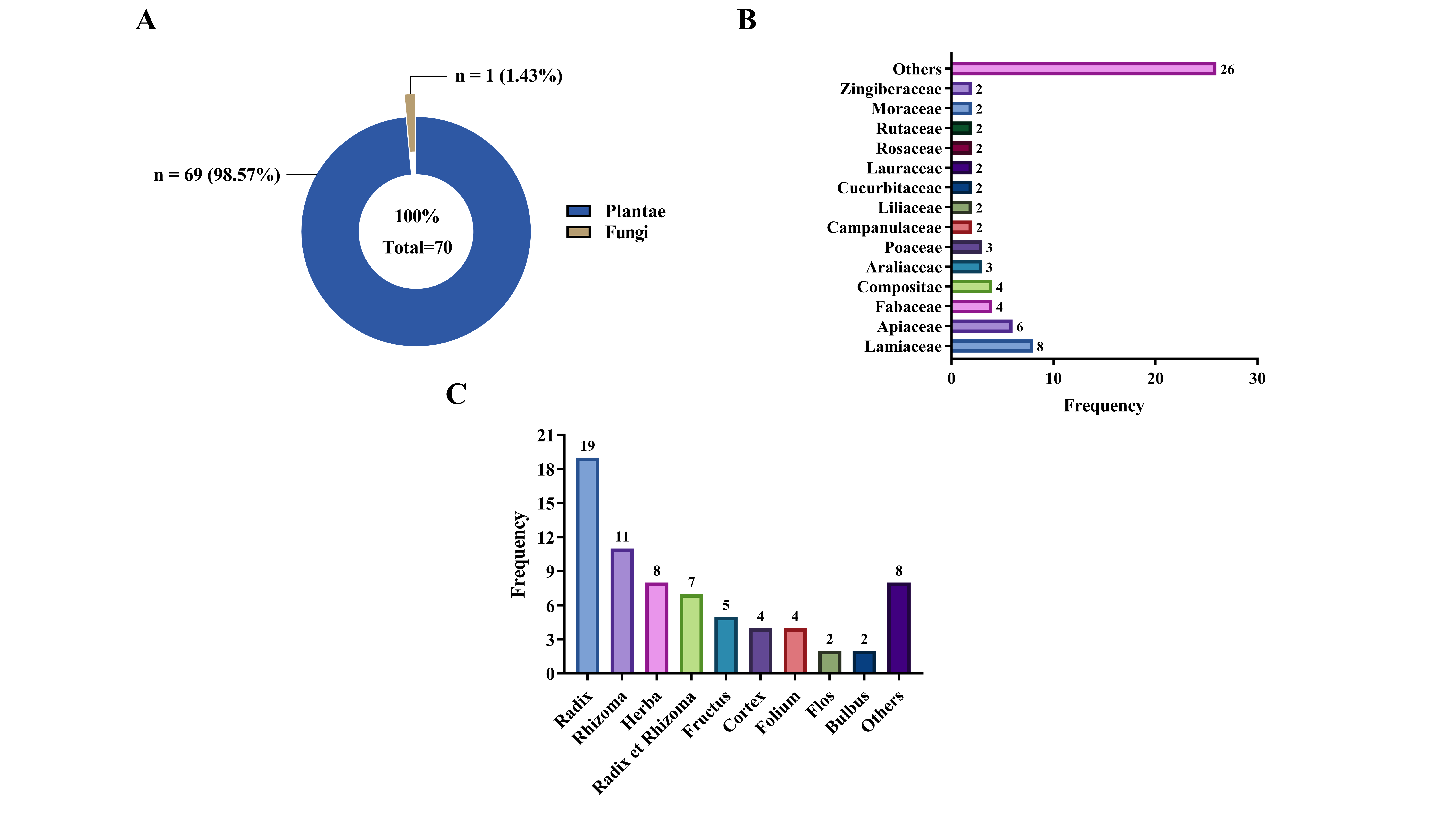 |
| --- |
| **Figure S3. Basic Information on 70 Medicinal Materials against COVID-19 (A) Kingdom, (B) Family, and (C) Part Used**  **Note: Family and Part Used with frequency = 1 are merged into “Others”** |
